# Genetic Variants of *APOL1* Are Major Determinants of Kidney Failure in People of African Ancestry With HIV

**DOI:** 10.1016/j.ekir.2022.01.1054

**Published:** 2022-01-25

**Authors:** Rachel K.Y. Hung, Elizabeth Binns-Roemer, John W. Booth, Rachel Hilton, Mark Harber, Beatriz Santana-Suarez, Lucy Campbell, Julie Fox, Andrew Ustianowski, Catherine Cosgrove, James E. Burns, Amanda Clarke, David A. Price, David Chadwick, Denis Onyango, Lisa Hamzah, Kate Bramham, Caroline A. Sabin, Cheryl A. Winkler, Frank A. Post, GEN-AFRICA Study Group, John Booth, Anele Waters, James Hand, Chris Clarke, Sarah Murphy, Maurice Murphy, Marion Campbell, Amanda Clarke, Celia Richardson, Alyson Knott, Gemma Weir, Rebecca Cleig, Helena Soviarova, Lisa Barbour, Tanya Adams, Vicky Kennard, Vittorio Trevitt, Rachael Jones, Jeremy Levy, Alexandra Schoolmeester, Serah Duro, Rachel Hilton, Julie Fox, May Rabuya, Lisa Hamzah, Deborah Jordan, Teresa Solano, Hiromi Uzu, Karen Williams, Julianne Lwanga, Linda Ekaette Reid-Amoruso, Hannah Gamlen, Robert J. Stocker, Fiona Ryan, Anele Waters, Karina Mahiouz, Tess Cheetham, Claire Williams, Achyuta Nori, Caroline Thomas, Sivaraj Venkateshwaran, Jessica Doctor, Andrea Berlanga, Frank Post, Beatriz Santana-Suarez, Leigh McQueen, Priya Bhagwandin, Lucy Campbell, Bee Barbini, Emily Wandolo, Tim Appleby, Deborah Jordan, Lois Driver, Sophy Parr, Hongbo Deng, Julie Barber, Andrew Crowe, Chris Taylor, Mary Poulton, Vida Boateng, Marie-Pierre Klein, Caitlin O'Brien, Samuel Ohene-Adomako, Christian Buckingham, Daniel Trotman, Killian Quinn, Kate Flanagan, Verity Sullivan, Holly Middleditch, Itty Samuel, Elizabeth Hamlyn, Candice McDonald, Ana Canoso, Emeka Agbasi, Maria Liskova, Sarah Barber, Amanda Samarawickrama, Zoe Ottaway, Claire Norcross, Amelia Oliveira, Kate Bramham, Jane Minton, Gary Lamont, Ruby Cross, Gaushiya Saiyad, Shadia Ahmed, Rebecca Ashworth, Nicola Window, J. Murira, Khine Phyu, Andrew Ustianowski, Gabriella Lindergard, Jonathan Shaw, Sarah Holland, Claire Fox, Jan Flaherty, Margaret-Anne Bevan, Valerie George, David Chadwick, Marie Branch, Pauline Lambert, Adele Craggs, Sarah Pett, Hinal Lukha, Nina Vora, Marzia Fiorino, Maria Muller Nunez, Deirdre Sally, James E. Burns, Erica Pool, Rebecca Matthews, David Ashley Price, Tara Stothard, Bijal Patel, Ian McVittie, Ciara Kennedy, Uli Shwab, Brendan Payne, Sarah Duncan, Jill Dixon, Mathias Schmid, Adam Evans, Christopher Duncan, Ewan Hunter, Yusri Taha, Natasha Astill, Cheryl Winkler, Elizabeth Binns-Roemer, Victor David, Jonathan Ainsworth, Rachel Vincent, Stephen Kegg, Chloe Saad, Sarah Skinner, Hocine Azzoug, Judith Russell, Tarik Moussaoui, Celia Richardson, Emily Mabonga, Donna Ward, J. Francoise, W. Larbi, Sue Mitchell, A. Manning, V. Russell, Fiona Burns, Mark Harber, Nnenna Ngwu, Jonathan Edwards, Nargis Hemat, Tom Fernandez, Filippo Ferro, Jorge Ferreira, Alice Nightingale, Tasha Oakes-Monger, Darwin Matila, Pedro Nogueira, Victoria Mutagwanya, Catherine Cosgrove, Lisa Hamzah, Catherine Emily Isitt, Helen Webb, Joyce Popoola, Kate Korley, Mark Mencias, Patricia Ribeiro, Rajeshwar Ramkhelawn, Sandra Oliva Lara, Sara Sajijad, Alan Winston, Jeremy Levy, Amber Shaw, Claire Petersen, Kyle Ring, Melanie Rosenvinge, Chloe Saad, Sarah Skinner, Thembi Moyo, Faith Odong, Katherine Gantert, Tina Ibe, Denis Onyango, Caroline Sabin, Teresa Hill

**Affiliations:** 1 King’s College London, London, UK; 2 Basic Research Laboratory, Frederick National Laboratory for Cancer Research and the National Cancer Institute, Frederick, Maryland, USA; 3 Barts Health NHS Trust, London, UK; 4 Guy’s and St Thomas’ NHS Foundation Trust, London, UK; 5 Royal Free London Hospital NHS Foundation Trust, London, UK; 6 Pennine Acute Hospitals NHS Foundation Trust, Manchester, UK; 7 St George’s Hospital NHS Foundation Trust, London, UK; 8 University College London, London, UK; 9 Central and North West London NHS Foundation Trust, London, UK; 10 Brighton and Sussex University Hospital NHS Trust, Brighton, UK; 11 Brighton and Sussex Medical School Department of Infectious Disease, Brighton, UK; 12 The Newcastle Upon Tyne Hospitals, Newcastle, UK; 13 South Tees Hospitals NHS Foundation Trust, Middlesbrough, UK; 14 Africa Advocacy Foundation, London, UK; 15 King’s College Hospital NHS Foundation Trust, London, UK

**Keywords:** Africa, APOL1, diaspora, HIV, HIVAN, kidney

## Abstract

**Introduction:**

Variants of the *APOL1* gene are associated with chronic kidney disease (CKD) in people of African ancestry, although evidence for their impact in people with HIV are sparse.

**Methods:**

We conducted a cross-sectional study investigating the association between *APOL1* renal risk alleles and kidney disease in people of African ancestry with HIV in the UK. The primary outcome was end-stage kidney disease (ESKD; estimated glomerular filtration rate [eGFR] of <15 ml/min per 1.73 m^2^, chronic dialysis, or having received a kidney transplant). The secondary outcomes included renal impairment (eGFR <60 ml/min per 1.73 m^2^), albuminuria (albumin-to-creatinine ratio [ACR] >30 mg/mmol), and biopsy-proven HIV-associated nephropathy (HIVAN). Multivariable logistic regression was used to estimate the associations between *APOL1* high-risk genotypes (G1/G1, G1/G2, G2/G2) and kidney disease outcomes.

**Results:**

A total of 2864 participants (mean age 48.1 [SD 10.3], 57.3% female) were genotyped, of whom, 354 (12.4%) had *APOL1* high-risk genotypes, and 99 (3.5%) had ESKD. After adjusting for demographic, HIV, and renal risk factors, individuals with *APOL1* high-risk genotypes were at increased odds of ESKD (odds ratio [OR] 10.58, 95% CI 6.22–17.99), renal impairment (OR 5.50, 95% CI 3.81–7.95), albuminuria (OR 3.34, 95% CI 2.00–5.56), and HIVAN (OR 30.16, 95% CI 12.48–72.88). An estimated 49% of ESKD was attributable to *APOL1* high-risk genotypes.

**Conclusion:**

*APOL1* high-risk genotypes were strongly associated with kidney disease in people of African ancestry with HIV and accounted for approximately half of ESKD cases in this cohort.

## Graphical abstract

**Figure:**
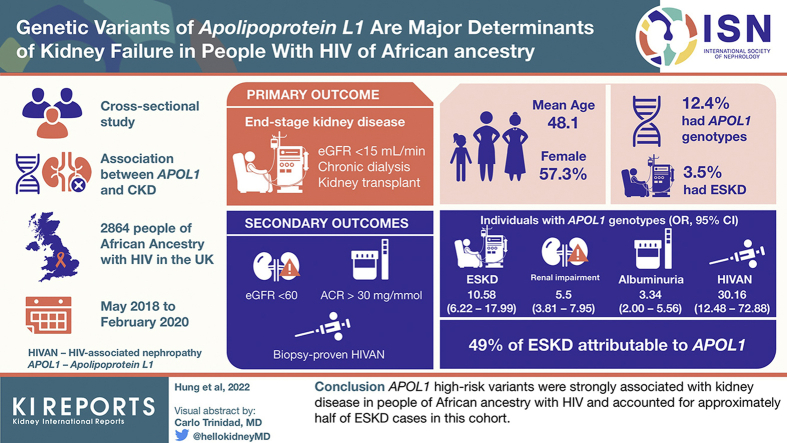

People of African ancestry are at approximately 3-fold increased risk of developing kidney failure requiring renal replacement therapy (RRT). African American and Black British people constitute 13.4% and 3% of the USA and UK general population, respectively, but 31.5% and 7.8% of patients requiring RRT in these countries, respectively, are of African ancestry.^1^^,^^2^ Hypertension and diabetes mellitus are important risk factors for kidney disease and are highly prevalent in people of African ancestry.^3^^,^^4^ However, homozygosity or compound heterozygosity for G1 and G2 variants of the *APOL1* gene on human chromosome 22, which are exclusively found in people of recent African ancestry, in whom they confer protection against *Trypanosoma brucei gambiense* (G1 variant) and *Trypanosoma* *brucei rhodesiense* (G2 variant) infections, have been identified as a potent risk factor for nondiabetic CKD.^5^, ^6^, ^7^ The prevalence of *APOL1* variants varies widely by African geographic region. The highest rates of *APOL1* high-risk genotypes have been reported in West Africans (30%–40% in Igbo- and Yoruba-speaking Nigerians and Asante-speaking Ghanaians), with substantially lower rates (5%–12%) in populations from South and East Africa and almost complete absence in those from the horn of Africa (Ethiopia).^8^^,^^9^ Data from Europe showed that *APOL1* high-risk genotypes were also prevalent in people from the Caribbean.^10^ Hence, the prevalence of CKD in black populations varies substantially by region of ancestry.^11^

The main kidney pathologies associated with *APOL1* variants, especially in those with *APOL1* high-risk genotypes (G1/G1, G1/G2, G2/G2), are focal and segmental glomerulosclerosis (FSGS),^12^ focal global glomerulosclerosis (or solidified glomerulosclerosis), and collapsing glomerulopathy, particularly in those who have received interferon therapy,^13^ and more recently, in association with SARS-CoV-2 infection.^14^, ^15^, ^16^

HIVAN is a collapsing glomerulopathy and a major cause of kidney failure in populations with untreated or under-treated HIV infection, especially in those with *APOL1* high-risk genotypes.^15^^,^^16^ About 70% of individuals with HIVAN have *APOL1* high-risk genotypes, and these high-risk genotypes are strongly associated with HIVAN (OR 29–89) in case-control studies.^12^^,^^17^ It has been estimated that, in the absence of effective antiretroviral therapy (ART), those with *APOL1* high-risk genotypes have a 50% life-time risk of developing HIVAN.^18^ Immunodeficiency, reflected by a low (nadir) CD4 cell count, is an additional risk factors for HIVAN, severe CKD, and kidney disease progression in people of African ancestry with HIV.^19^, ^20^, ^21^

Previous studies in adult populations of African Americans, Nigerians, and South Africans with HIV have also reported associations between *APOL1* high-risk genotypes and FSGS,^22^ albuminuria,^23^^,^^24^ proteinuria, decline in eGFR,^25^ eGFR <60 ml/min per 1.73 m^2^,^26^ and CKD,^27^ and raised the possibility that individuals with a single *APOL1* variant may be at increased risk of developing kidney disease^17^^,^^26^ and that the G1 variant may pose greater renal risk than the G2 variant.^12^^,^^17^ Although participants with kidney failure (ESKD) were included in some of these studies, the association between *APOL1* variants and ESKD has not been studied in people with HIV, and the burden of ESKD that is attributable to *APOL1* variants remains unknown, particularly in geographically diverse African populations. Hence, we established a large cohort of people of recent African ancestry with HIV in the United Kingdom to study the prevalence of *APOL1* risk alleles and their relationship to kidney failure requiring RRT, HIVAN/FSGS, and milder manifestations of CKD.

## Methods

The “Genetic Markers of Kidney Disease Progression in People of African Ancestry with HIV in the United Kingdom (GEN-AFRICA) study” enrolled individuals of black ethnicity aged 18 years or more at 15 HIV clinics and 3 dialysis/kidney transplantation centers across England between May 2018 and February 2020. During a single study visit, informed consent was obtained, and demographic data including country of birth of both parents and clinical information were collected from participants using questionnaires corroborated through review of clinical records. Laboratory data, including nadir and most recent CD4 cell count, viral hepatitis status, and HIV viral load, were obtained from electronic patient records. Renal function was assessed by measuring serum creatinine and urine protein-to-creatinine ratio (PCR) in local laboratories, and ACR in stored urine samples in a central laboratory. The study was approved by an NHS Research Ethics Committee and Health Research Authority (18/LO/0234 and 239895).

For *APOL1* genetic analysis, DNA was extracted from buffy coats using Qiagen extraction kits. *APOL1* variant sites were genotyped using custom TaqMan assays (Thermo Fisher Scientific, Waltham, MA): rs73885319 (Assay ID-AH20SD1), rs60910145 (Assay ID-AHWR1JA), and rs71785313 (Assay ID-AH1RT7T). These assays have been validated by Sanger sequencing,^28^^,^^29^ and the primers have been designed specifically to avoid confounding by the insertion and deletion (G2) site.^30^ High-risk genotypes were defined as those containing 2 high-risk alleles (G1/G1, G1/G2, or G2/G2); low-risk genotypes were defined as those containing 0 (G0/G0) or 1 risk allele (G0/G1, G0/G2) haplotypes. The exposure in the present analysis was *APOL1* risk allele status comparing high-risk with low-risk genotypes.

Participants were grouped by region of African ancestry based on country of birth of both parents: East, South, Central, and West Africa as defined by the African Union,^31^ with the exception of Angola, which was included in the Central rather than South region, or the Caribbean. Participants whose parents’ country of birth was unknown, of different African regions or outside sub-Saharan Africa or the Caribbean, and those of mixed African/Caribbean ancestry were grouped together as “Other.” eGFR was calculated using the CKD Epidemiology Collaboration equation^32^ with application of the correction factor for black ethnicity. Participants were stratified by eGFR based on Kidney Disease: Improving Global Outcomes CKD guidelines^33^ and those with eGFR <15 ml/min per 1.73 m^2^, a kidney transplant, or receiving chronic dialysis were categorized as having ESKD, the primary outcome.

Renal biopsy reports were reviewed and adjudicated by a renal physician (JB) and, in case of discrepancy, a histopathologist to identify cases of HIVAN, (primary) FSGS, and arterionephrosclerosis.^20^ A clinical case definition of HIVAN/FSGS/hypertensive nephropathy was applied to participants with stage 4/5 CKD who lacked renal histology comprising all the following criteria at CKD diagnosis: (i) renal impairment (eGFR <60 ml/min per 1.73 m^2^ including those with ESKD), (ii) urine PCR >100 mg/mmol, (iii) no diabetes, (iv) echogenic and/or normal or enlarged kidneys on ultrasound, and (v) lack of an alternative diagnosis.^34^ Diabetes mellitus and hypertension were predominantly self-reported diagnoses; medical records were reviewed for those reporting but not on treatment for these conditions to verify the diagnosis. In addition, diabetes cases were ascertained through review of medical records of those with glycosuria.

### Statistical Methods

Baseline characteristics of the study population, stratified by *APOL1* status and by ESKD status and by eGFR (> or <60 ml/min per 1.73 m^2^), were compared using χ^2^ for categorical variables and Kruskal–Wallis tests or analysis of variance for continuous variables, as appropriate. After confirming that there was no difference in effect estimates of the outcomes for those with 0 and 1 *APOL1* variants, these individuals were combined as the reference low-risk group. Logistic regression was used to describe the association between *APOL1* high-risk genotypes and kidney disease status; likelihood ratio tests were used to assess the strength of association at each level.

It was decided *a priori* to include age and sex in all models; models for the primary outcome were additionally adjusted for region of ancestry, HIV factors (time since HIV diagnosis, prior AIDS, current CD4 cell count, hepatitis C [anti–hepatitis C virus]), and renal factors (diabetes mellitus and cardiovascular disease [a composite of any history of myocardial infarction, coronary artery disease, peripheral vascular disease, stroke, heart failure and cardiomyopathy]). We also analyzed the association between *APOL1* high-risk genotypes and the following secondary outcomes: (i) eGFR <60 ml/min per 1.73 m^2^, (ii) proteinuria (PCR >100 mg/mmol, excluding those with ESKD), (iii) albuminuria (ACR >30 mg/mmol, excluding those with ESKD), (iv) biopsy-confirmed HIVAN or FSGS, and (v) clinical case definition of HIVAN/FSGS/hypertensive nephropathy. To further characterize the association between *APOL1* status and ESKD, we performed analyses to determine the association of each of the high-risk genotypes with ESKD/renal impairment, with different low-risk genotypes as referent (G0/G0 only and in combination with G1/G0 or G2/G0). We also investigated the associations between carriage of a single G1 or G2 allele (G1/G0 and G2/G0 vs G0/G0) and ESKD/renal impairment. Finally, we estimated the population of ESKD/renal impairment attributable to *APOL1* high-risk genotypes as population attributable risk = P_e_ (RR_e_−1)/1+P_e_ (RR_e_−1) × 100, where P_e_ was the prevalence of the exposure and RR_e_ was the relative risk of the disease because of the exposure.^35^^,^^36^

As hypertension is almost invariably present in individuals with advanced CKD, the main analyses were not adjusted for hypertension. Supplementary analyses were performed to describe the relationship between *APOL1* high-risk genotypes and hypertension in participants with eGFR ≥90, 60 to 90, and <60 ml/min per 1.73 m^2^, and to assess the effect of including hypertension on the association between *APOL1* high-risk genotypes and ESKD/renal impairment. Further sensitivity analyses with exclusion of the correction for ethnicity from the eGFR calculations were performed. All statistical analyses were done using STATA v16 (StataCorp, College Station, TX).

## Results

A total of 3027 individuals were enrolled in the GEN-AFRICA study; *APOL1* genotyping was successful for 2864 (94.6%); 5.4% failed to provide reliable allele calls because of low quality or quantity of DNA. The *APOL1* status was G0/G0 in 1406 (49.1%), G0/G1 or G0/G2 in 1104 participants (38.5%), and 354 (12.4%) had high-risk genotypes. The overall allele frequencies for G0, G1, and G2 were 68.4%, 19.8%, and 11.9%, respectively. The demographic and clinical characteristics of the participants stratified by *APOL1* status are shown in Table 1. Participants had a mean age of 48.1 (SD 10.3) years and were predominantly of East (19.3%), South (26.7%), and West African (30.0%) ancestry, with a further 12% of Caribbean ancestry. Most participants had long-standing (mean 14.0 years) and well-controlled HIV (93.2% had a viral load <200 copies/ml). Participants with *APOL1* high-risk genotypes had lower eGFR and higher levels of proteinuria and albuminuria and were more likely to have hypertension. Participants with 0 and 1 *APOL1* risk haplotypes were indistinguishable in terms of eGFR (*P* = 0.21), PCR (*P* = 0.27), and ACR (*P* = 0.29). The prevalence of diabetes and cardiovascular disease was similar across all 3 groups.

**Table 1:** Baseline characteristics of study participants stratified by *APOL1* status

Participant characteristics		Total *N* = 2864	Number of *APOL1* variants			*P* value
			0	1	2	
			*n* = 1406	*n* = 1104	*n* = 354	
Age, yr	mean (SD)	48.1 (10.3)	48.1 (10.3)	48.1 (10.4)	48.2 (10.0)	0.97
Sex, female	*n* (%)	1641 (57.3)	833 (59.3)	622 (56.3)	186 (52.5)	0.05
Region of African ancestry						<0.001
East Africa	*n* (%)	554 (19.3)	432 (30.7)	111 (10.1)	11 (3.1)	
South Africa	*n* (%)	765 (26.7)	421 (29.9)	292 (26.4)	52 (14.7)	
Central Africa	*n* (%)	157 (5.5)	90 (6.4)	58 (5.3)	9 (2.5)	
West Africa	*n* (%)	859 (30.0)	237 (16.9)	403 (36.5)	219 (61.9)	
Caribbean	*n* (%)	347 (12.1)	134 (9.5)	163 (14.8)	50 (14.1)	
Other	*n* (%)	182 (6.4)	92 (6.5)	77 (7.0)	13 (3.7)	
HIV mode of acquisition						0.04
Heterosexual	*n* (%)	2347 (81.9)	1170 (83.2)	890 (80.6)	287 (81.1)	
MSM	*n* (%)	74 (2.6)	36 (2.6)	27 (2.4)	11 (3.1)	
Vertical	*n* (%)	232 (8.1)	90 (6.4)	115 (10.4)	27 (7.6)	
Blood products	*n* (%)	23 (0.8)	14 (1.0)	6 (0.5)	3 (0.8)	
Unknown	*n* (%)	188 (6.6)	96 (6.8)	66 (6.0)	26 (7.3)	
Time since HIV diagnosis, years	mean (SD)	14.0 (6.5)	14.5 (6.6)	13.7 (6.3)	12.8 (6.4)	<0.001
Previous AIDS	*n* (%)	665 (24.0)	333 (24.5)	246 (22.9)	86 (25.1)	0.59
Nadir CD4 cell count, cells/mm^3^	median (IQR)	200 (80–340)	206 (90–337)	203 (71–346)	185 (70–315)	0.41
Recent CD4 cell count, cells/mm^3^	median (IQR)	560 (403-735)	569 (408-744)	558 (400-738)	542 (388-703)	0.14
On antiretroviral therapy	*n* (%)	2832 (98.9)	1390 (98.9)	1095 (99.2)	347 (98.0)	0.19
HIV RNA <200 copies/ml	*n* (%)	2669 (93.2)	1326 (94.3)	1016 (92.0)	327 (92.4)	0.06
HBsAg positive	*n* (%)	159 (5.6)	63 (4.5)	72 (6.6)	24 (6.9)	0.05
Anti-HCV positive	*n* (%)	35 (1.2)	13 (0.9)	18 (1.7)	4 (1.2)	0.28
Diabetes	*n* (%)	286 (10.1)	157 (11.3)	95 (8.7)	34 (9.7)	0.10
Hypertension	*n* (%)	915 (32.0)	398 (28.3)	342 (31.0)	175 (49.4)	<0.001
Cardiovascular disease^a^	*n* (%)	123 (4.3)	56 (4.0)	46 (4.2)	21 (5.9)	0.26
BMI, kg/m^2^						0.07
<18.5	*n* (%)	23 (0.8)	9 (0.7)	14 (1.3)	0 (0.0)	
18.5–24.9	*n* (%)	630 (22.4)	328 (23.8)	237 (21.7)	65 (18.8)	
25–29.9	*n* (%)	1016 (36.1)	484 (35.1)	393 (36.1)	139 (40.3)	
>30	*n* (%)	1146 (40.7)	559 (40.5)	446 (40.9)	141 (40.9)	
Smoking status						
Never	*n* (%)	2218 (77.4)	1078 (76.7)	852 (77.2)	288 (81.4)	
Ex	*n* (%)	321 (11.2)	168 (11.9)	122 (11.1)	31 (8.8)	
Current	*n* (%)	325 (11.3)	160 (11.4)	130 (11.8)	35 (9.9)	
eGFR,^b^ ml/min per 1.73 m^2^	median (IQR)	99 (83–116)	102 (86–118)	100 (84–115)	87 (60–106)	<0.001
>90	*n* (%)	1848 (64.5)	956 (68.0)	730 (66.1)	162 (45.8)	<0.001
60–89	*n* (%)	795 (27.8)	372 (26.5)	317 (28.7)	106 (29.9)	
30–59	*n* (%)	106 (3.7)	47 (3.3)	34 (3.1)	25 (7.1)	
15–29	n (%)	16 (0.6)	5 (0.4)	5 (0.5)	6 (1.7)	
<15 (ESKD^c^)	n (%)	99 (3.5)	26 (1.8)	18 (1.6)	55 (15.5)	
Urine PCR,^d^ mg/mmol	median (IQR)	8.6 (6 - 13.5)	8.6 (6 - 13.7)	8.5 (6 - 13.2)	9 (6 -14.9)	0.13
<15	*n* (%)	2193 (79.3)	1085 (78.6)	879 (80.9)	229 (76.6)	0.36
15–49	*n* (%)	431 (15.6)	223 (16.2)	159 (14.6)	49 (16.4)	
50–99	*n* (%)	74 (2.7)	37 (2.7)	28 (2.6)	9 (3.0)	
≥100	*n* (%)	67 (2.4)	35 (2.5)	20 (1.8)	12 (4.0)	
Urine ACR,^d^ mg/mmol	median (IQR)	0.7 (0.4–1.9)	0.8 (0.4–1.7)	0.7 (0.4–1.7)	1 (0.5–3)	<0.001
<3	*n* (%)	2189 (82.2)	1103 (82.7)	868 (83.7)	218 (74.4)	<0.001
3–30	*n* (%)	375 (14.1)	181 (13.6)	142 (13.7)	52 (17.7)	
>30	*n* (%)	99 (3.7)	49 (3.7)	27 (2.6)	23 (7.8)	

ACR, urine albumin-to-creatinine ratio; BMI, body mass index; CKD-EPI, Chronic Kidney Disease-Epidemiology Collaboration; eGFR, estimated glomerular filtration rate; ESKD, end-stage kidney disease; HBsAg, hepatitis B surface antigen; HCV, hepatitis C virus; IQR, interquartile range; MSM, men who have sex with men; PCR, urine protein-to-creatinine ratio.

a Cardiovascular disease = composite of any previous history of myocardial infarction, coronary artery disease, peripheral vascular disease, stroke, heart failure, and cardiomyopathy.

b eGFR calculated with CKD-EPI formula with correction of black ethnicity included.

c ESKD = eGFR <15ml/min per 1.73 m^2^, dialysis for over 3 months, or having had a kidney transplant.

d PCR and ACR do not include participants with ESKD.

**Figure 1:**
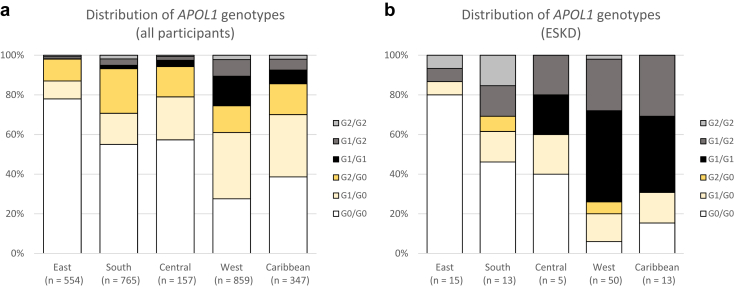
Distribution of *APOL1* alleles by region of African ancestry, in (a) all participants and (b) those with ESKD. East, South, Central, and West refer to regions within sub-Saharan Africa; ESKD includes participants with eGFR. Numerical data are shown in Supplementary Table S7. eGFR, estimated glomerular filtration rate; ESKD, end-stage kidney disease.

**Figure 2:**
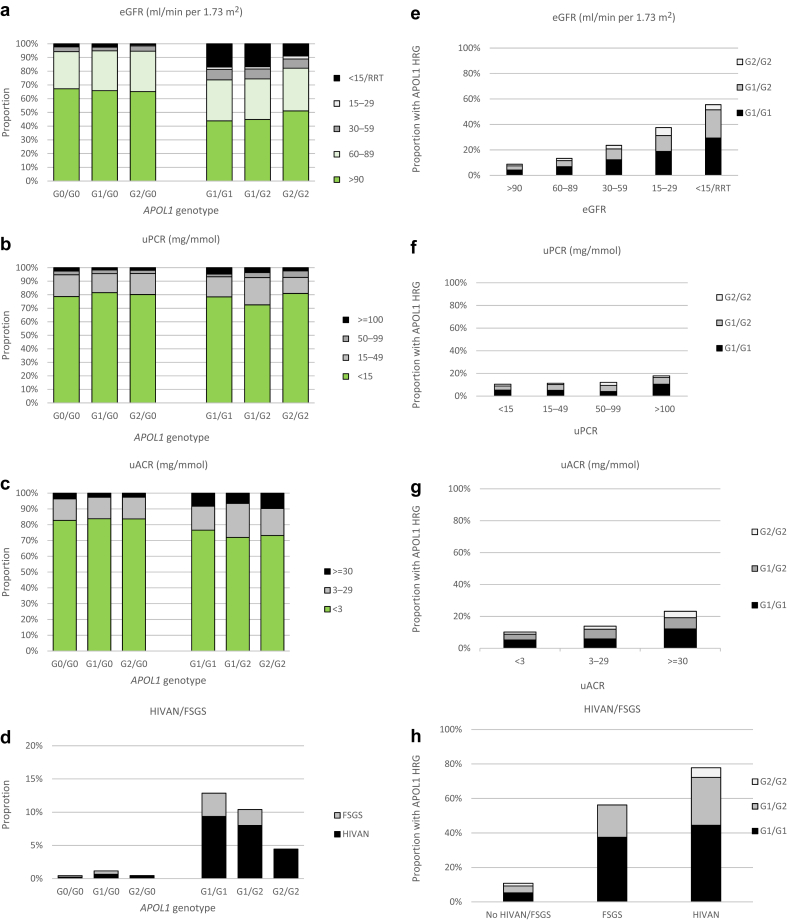
Kidney function, kidney pathology and *APOL1* genotype. Distribution of (a) eGFR, (b) uPCR, (c) uACR, and (d) cases of HIVAN/FSGS by APOL1 status, and prevalence of APOL1 high-risk genotypes by (e) eGFR, (f) PCR, (g) ACR categories, and (h) HIVAN/FSGS status. eGFR, estimated glomerular filtration rate; FSGS, focal and segmental glomerulosclerosis; HIVAN, HIV-associated nephropathy; RRT, renal replacement therapy; uACR, urine albumin-to-creatinine ratio; uPCR, urine protein-to-creatinine ratio.

Figure 1a and b shows the distribution of *APOL1* risk haplotypes across the regions of ancestry. About 60% of participants of West African and Caribbean ancestry carried at least 1 *APOL1* variant, compared with 40% of those of South and Central African and 20% of those of East African ancestry (with notable absence of *APOL1* risk variants in those of Ethiopian and Eritrean ancestry; Supplementary Figure S1). *APOL1* high-risk genotypes were present in >10% of participants of West African (especially in those from Nigeria, Ghana, and Cote d’Ivoire; Supplementary Figure S1) and Caribbean ancestry. G1 was the predominant variant in West Africans and Caribbeans while G2 predominated in East and South Africans. Across all regions, *APOL1* high-risk genotypes were more common among individuals with ESKD, reaching a prevalence of 69% to 74% in Caribbean and West African participants. Among participants who had undergone renal biopsy, HIVAN/FSGS was the predominant kidney disease etiology in those of West, Central, South African, or Caribbean ancestry; HIVAN/FSGS was notably uncommon among those of East African ancestry (Supplementary Figure S2). Figure 2 shows the distribution of eGFR (Figure 2a), PCR (Figure 2b), and ACR (Figure 2c) and the prevalence of HIVAN/FSGS (Figure 2d) by *APOL1* genotype and the prevalence of *APOL1* high-risk genotypes in participants stratified by these markers/pathologies (Figure 2e-h). Participants with 0 and 1 risk genotypes had similar eGFR, PCR, and ACR, and few had biopsy-confirmed HIVAN/FSGS. By contrast, renal impairment, ESKD, and HIVAN/FSGS were all considerably more common in those with high-risk genotypes. Nonetheless, about 70% to 80% of participants with high-risk genotypes had eGFR ≥60 ml/min per 1.73 m^2^ and/or normal urine PCR or ACR. The proportion of participants with high-risk genotypes increased from 9% among those with eGFR >90 ml/min per 1.73 m^2^ to 55% to 57% among those with ESKD and FSGS, and to 78% in those with HIVAN. Compared with those with G1/G1 and G1/G2 genotypes, a smaller proportion of individuals with G2/G2 genotypes had renal impairment (9.3%, *P* = 0.24), ESKD (7.3%, *P* = 0.17), and HIVAN/FSGS (5.4%, *P* = 0.41); however, these differences were not statistically significant.

A total of 99 participants had ESKD, of whom, 55 (55.6%) had *APOL1* high-risk genotypes, and 63 (63.6%) were of West African or Caribbean ancestry. Participants with ESKD were older, less likely to be female, and more likely to have had an AIDS-defining illness and developed diabetes or cardiovascular disease; nadir and recent CD4 cell counts were lower, and almost all (94.9%) had hypertension. A similar pattern was observed for the comparison of those with eGFR less or greater than 60 ml/min per 1.73 m^2^ (Supplementary Table S1). We further explored the relationship between *APOL1* status, eGFR, and hypertension and found no association between *APOL1* high-risk genotypes (vs. 0/1 risk genotypes) and hypertension in 1848 participants with eGFR >90 ml/min per 1.73 m^2^ and evidence for a graded association in those with reduced eGFR (Supplementary Table S2).

In univariable analysis, *APOL1* high-risk genotypes, West African ancestry, age, sex, prior AIDS, lower CD4 cell count, HIV viral load, anti– hepatitis C virus, diabetes, hypertension, and cardiovascular disease were associated with ESKD. In multivariable analysis, *APOL1* high-risk genotypes remained strongly associated with ESKD (adjusted OR 10.58, 95% CI 6.22–17.99) (Table 2). West African ancestry and demographic factors were no longer associated with ESKD after adjustment for *APOL1* status, whereas low CD4 cell count, diabetes, and cardiovascular disease remained associated with ESKD. Similar, albeit weaker, associations were observed between *APOL1* high-risk genotypes and albuminuria or renal impairment (Table 2 and Supplementary Table S3A-G). Strong associations (OR >10) were also observed between *APOL1* high-risk genotypes and biopsy-confirmed FSGS, a clinical diagnosis of HIVAN/FSGS/hypertensive nephropathy, and biopsy-confirmed HIVAN (OR 30.16, 95% CI 12.48–72.88). Additional analyses showed that each of the high-risk genotypes (G1/G1, G1/G2, G2/G2) was associated with ESKD and renal impairment (Supplementary Table S4). No significant associations between G1/G0 or G2/G0 (vs. G0/G0) and either ESKD or renal impairment were observed (G1: OR 1.08 [95% CI 0.49–2.40] for ESKD and OR 0.72 [0.44–1.18] for renal impairment; G2: OR 0.34 [0.09–1.25] for ESKD and OR 0.73 [0.42–1.28] for renal impairment) (Supplementary Table S4). The proportion of ESKD and renal impairment attributable to *APOL1* high-risk genotypes (population attributable risk) was 49.4% and 30.4%, respectively.

**Table 2:** Associations between *APOL1* high-risk genotypes and renal outcomes

Participant characteristics	*N*	Univariable			Multivariable		
		OR	95% CI	*P* value	OR	95% CI	*P* value
Primary outcome							
End-stage kidney disease	99	10.31	6.81–15.60	<0.001	10.58	6.22–17.99	<0.001^a^
Secondary outcomes							
Proteinuria (PCR >100 mg/mmol)	67	1.83	0.97–3.46	0.06			
Albuminuria (ACR >30 mg/mmol)	99	2.57	1.59–4.17	<0.001	3.34	2.00–5.56	<0.001^b^
eGFR <60 ml/min per 1.73 m^2^	221	5.65	4.19–7.61	<0.001	5.50	3.81–7.95	<0.001^c^
FSGS/HIVAN/hypertensive nephropathy (clinical diagnosis)	19	14.64	5.46–39.27	<0.001	12.77	4.46–36.59	<0.001^d^
FSGS (biopsy confirmed)	15	11.81	4.17–33.39	<0.001	12.86	4.04–40.99	<0.001^d^
HIVAN (biopsy confirmed)	37	24.49	11.45–52.36	<0.001	30.16	12.48–72.88	<0.001^d^

ACR, urine albumin-to-creatinine ratio; CVD, cardiovascular disease; DM, diabetes mellitus; eGFR, estimated glomerular filtration rate; FSGS, focal segmental glomerulosclerosis; HCV, hepatitis C virus; HIVAN, HIV-associated nephropathy; OR, odds ratio; PCR, urine protein-to-creatinine ratio.

Models are adjusted for:

a Demographic (age, sex, and region of ancestry), HIV (AIDS, CD4 cell count), anti-HCV, DM, and CVD.

b Demographic (age, sex), HIV (Time since HIV diagnosis, AIDS, HIV RNA < 200 copies/ml), DM, and CVD.

c Demographic (age, sex, and region of ancestry), HIV (Time since HIV diagnosis, AIDS, CD4 cell count), anti-HCV, DM, and CVD.

d Demographic (age and sex), HIV (AIDS, CD4 cell count), anti-HCV, DM, and CVD.

We also analyzed the associations between *APOL1* high-risk genotypes and ESKD/renal impairment in models that included hypertension; these also yielded similar, albeit somewhat lower, ORs (Supplementary Table S5) and with exclusion of the correction for ethnicity from the eGFR calculations (Supplementary Table S6).

## Discussion

In this large African diaspora cohort of people with HIV living in the United Kingdom, *APOL1* high-risk genotypes were strongly associated with ESKD, renal impairment, and HIVAN/FSGS. Across the eGFR spectrum, the frequency of *APOL1* high-risk genotypes progressively increased from 9% in those with normal kidney function to 56% in those with ESKD. We observed no association between a single *APOL1* risk allele and either ESKD or renal impairment. Our data suggest that 30% of CKD and 49% of ESKD in this population of sub-Saharan African ancestry with HIV may be attributable to *APOL1* high-risk genotypes, with the greatest burden among those of West African ancestry.

There are few studies that have reported on the relationship between *APOL1* high-risk genotypes and CKD in people with HIV. Two recent studies from Nigeria, in which most participants had well-controlled HIV, found a relatively weak (OR 2) association between *APOL1* high-risk genotypes and CKD (with or without renal impairment) or albuminuria.^23^^,^^27^ The large proportion of participants with ESKD among those with eGFR <60 ml/min per 1.73 m^2^ in our study may have contributed to the stronger association between *APOL1* high-risk genotypes and renal impairment. A previous study in African Americans reported no impact of *APOL1* status on the rate of progression to kidney failure requiring RRT in people who had been diagnosed with HIVAN.^22^ By contrast, *APOL1* high-risk genotypes were associated with a nearly 3-fold greater risk of kidney failure when other renal pathologies were present.^37^ The strong association between *APOL1* high-risk genotypes and ESKD in our study, in whom HIVAN was a common diagnosis, may reflect the inclusion of large numbers of participants with *APOL1* high-risk genotypes who had normal or only mildly impaired kidney function as compared with the case-only studies by Atta and Fine in which all participants had CKD.^22^^,^^37^

Consistent with 2 earlier studies,^12^^,^^17^ we report strong associations between *APOL1* high-risk genotypes and HIVAN. HIVAN is associated with advanced/uncontrolled HIV infection and the poorest renal outcomes.^38^, ^39^, ^40^, ^41^ Participants of West African ancestry were most likely to carry *APOL1* high-risk genotypes and were shown to have the highest likelihood of being diagnosed with HIVAN in an earlier analysis.^11^ Although it is unclear to what extent advanced CKD in our participants without kidney biopsies was due to HIVAN/FSGS or hypertension, the strong association between *APOL1* high-risk genotypes and our clinical case definition suggests that these etiologies may be relatively common, and that diabetic kidney disease and other causes of proteinuric renal failure not associated with *APOL1* risk alleles are relatively uncommon. The high burden of severe kidney disease (especially HIVAN/FSGS) attributable to *APOL1* high-risk genotypes suggests that early HIV diagnosis and ART-initiation may be important strategies to reduce the risk of ESKD, particularly in people of West African and Caribbean ancestry. The factors associated with onset and progression of kidney disease in individuals with well-controlled HIV who have *APOL1* high-risk genotypes require further study. *APOL1* high-risk genotypes have been associated with proteinuria and/or albuminuria in people with and without HIV.^23^^,^^42^, ^43^, ^44^, ^45^, ^46^ In our study, *APOL1* high-risk genotypes were more strongly associated with renal impairment (and ESKD) than with albuminuria or proteinuria. Most of our study participants had well-controlled HIV, and previous reports have suggested that HIVAN may remit after the introduction of ART.^47^ It is possible that a variable degree of kidney damage occurred in our participants with *APOL1* high-risk genotypes before HIV diagnosis and/or before initiation of ART, with subsequent stabilization or remission of kidney disease, resulting in persistent reductions in eGFR despite resolution of albuminuria and proteinuria. Transient rebound viraemia due to ART interruptions before study enrolment may have further contributed to this phenomenon. Of note, the prevalence of albuminuria in our participants was substantially lower than reported in the Nigerian population studied by Wudil *et al.*^23^ (17.8% vs. 39.9%), despite similar proportions of participants having suppressed HIV viral loads; most albuminuria was microalbuminuria and not associated with *APOL1* high-risk genotypes.

The somewhat stronger effect estimates of carriers of the G1 allele (G1/G1 or G1/G2) as compared with only G2 carriers (G2/G2) in those with *APOL1* high-risk genotypes are consistent with previous studies.^12^^,^^17^ However, we found no association between kidney disease outcomes in G1/G0 and G2/G0 carriers when compared with G0/G0 participants. This contrasts with the reported ORs of 2 and 10 of having HIVAN/FSGS in the Kopp *et al.*^12^ and Kasembeli *et al.*^17^ papers for carriers of a single G1 allele (G1/G0) but no association found for carriers of a single G2 allele (G2/G0). We were insufficiently powered to study the association between a single G1 or G2 variant and HIVAN/FSGS.

The strengths of this study are the large sample size including a substantial number of people with ESKD, broad geographic representation of the participants, and the healthcare setting which provides unrestricted access to ART and RRT. We acknowledge several limitations. First, the cross-sectional study design and use of a single creatinine reading to calculate eGFR may not truly reflect CKD status even though most participants were clinically well and recruited in an outpatient setting and likely to have had stable renal function. GFR estimates were calculated using the CKD Epidemiology Collaboration equation, which has not undergone rigorous validation in African populations. We did not include adjustment for specific ART drugs such as tenofovir disoproxil fumarate, atazanavir, and lopinavir, as these are generally avoided in people with or at risk of CKD.^19^^,^^20^ We also did not adjust for use of angiotensin converting enzyme inhibitors or angiotensin II receptor antagonists, which may have affected proteinuria and albuminuria measurements in some participants. We also lacked historical information on causes of CKD that are common in Africa, including infections such as schistosomiasis and malaria, heavy metal exposure, and use of medications such nonsteroidal anti-inflammatories or previous use of antimicrobials. Finally, the cohort was enriched for severe renal phenotypes, which may have affected the proportions of CKD and ESKD attributable to *APOL1* high-risk genotypes.

## Conclusion

This study shows that *APOL1* high-risk genotypes were strong predictors of ESKD in people of African ancestry with HIV, even within a healthcare setting with universal access to ART and in a population with mostly well-controlled HIV. *APOL1* high-risk genotypes accounted for almost half of ESKD and were particularly prevalent in people of West African and Caribbean ancestry. Early HIV diagnosis and initiation of ART are likely to be important strategies to reduce the risk of ESKD. Further investigation into other genetic and environmental factors that promote CKD in people of African ancestry with HIV is currently being undertaken, and this cohort is well placed to investigate the development and progression of kidney disease, especially in those with *APOL1* high-risk genotypes.

## Appendix

### List of GEN-AFRICA Study Group

Members of the Genetic markers of chronic kidney disease in people of African ancestry with HIV (GEN-AFRICA) Study Group

Barts Health NHS Trust, London (John Booth [PI], Anele Waters, James Hand, Chris Clarke, Sarah Murphy, Maurice Murphy); Brighton and Sussex University Hospitals, Brighton (Marion Campbell, Amanda Clarke [PI], Celia Richardson, Alyson Knott, Gemma Weir, Rebecca Cleig, Helena Soviarova, Lisa Barbour, Tanya Adams, Vicky Kennard, Vittorio Trevitt); Chelsea and Westminster Hospital, London (Rachael Jones [PI], Jeremy Levy, Alexandra Schoolmeester, Serah Duro); Guy’s and St Thomas’ Hospital, London (Rachel Hilton [PI], Julie Fox, May Rabuya, Lisa Hamzah, Deborah Jordan, Teresa Solano, Hiromi Uzu, Karen Williams, Julianne Lwanga, Linda Ekaette Reid-Amoruso, Hannah Gamlen, Robert J. Stocker, Fiona Ryan, Anele Waters, Karina Mahiouz, Tess Cheetham, Claire Williams, Achyuta Nori, Caroline Thomas, Sivaraj Venkateshwaran, Jessica Doctor, Andrea Berlanga); King’s College Hospital, London (Frank Post [CI], Beatriz Santana-Suarez, Leigh McQueen, Priya Bhagwandin, Lucy Campbell, Bee Barbini, Emily Wandolo, Tim Appleby, Deborah Jordan, Lois Driver, Sophy Parr, Hongbo Deng, Julie Barber, Andrew Crowe, Chris Taylor, Mary Poulton, Vida Boateng, Marie-Pierre Klein, Caitlin O'Brien, Samuel Ohene-Adomako, Christian Buckingham, Daniel Trotman, Killian Quinn, Kate Flanagan, Verity Sullivan, Holly Middleditch, Itty Samuel, Elizabeth Hamlyn, Candice McDonald, Ana Canoso, Emeka Agbasi, Maria Liskova, Sarah Barber, Amanda Samarawickrama, Zoe Ottaway, Claire Norcross, Amelia Oliveira, Kate Bramham); Leeds Teaching Hospitals NHS Trust, Leeds (Jane Minton [PI], Gary Lamont, Ruby Cross, Gaushiya Saiyad, Shadia Ahmed, Rebecca Ashworth, Nicola Window, J Murira, Khine Phyu); North Manchester General Hospital, Manchester (Andrew Ustianowski [PI], Gabriella Lindergard, Jonathan Shaw, Sarah Holland, Claire Fox, Jan Flaherty, Margaret-Anne Bevan, Valerie George); South Tees Hospitals NHS Foundation Trust, Middlesbrough (David Chadwick [PI], Marie Branch, Pauline Lambert, Adele Craggs); Mortimer Market Centre, Central and NorthWest London NHS Foundation Trust, London (Sarah Pett [PI], Hinal Lukha, Nina Vora, Marzia Fiorino, Maria Muller Nunez, Deirdre Sally, James E. Burns, Erica Pool, Rebecca Matthews); Newcastle upon Tyne Hospitals, Newcastle (David Ashley Price [PI], Tara Stothard, Bijal Patel, Ian McVittie, Ciara Kennedy, Uli Shwab, Brendan Payne, Sarah Duncan, Jill Dixon, Mathias Schmid, Adam Evans, Christopher Duncan, Ewan Hunter, Yusri Taha, Natasha Astill); National Cancer Institute, Frederick, USA (Cheryl Winkler, Elizabeth Binns-Roemer, Victor David); North Middlesex University Hospital, London (Jonathan Ainsworth, Rachel Vincent [PI]); Queen Elizabeth Hospital, Woolwich (Stephen Kegg [PI], Chloe Saad, Sarah Skinner, Hocine Azzoug, Judith Russell, Tarik Moussaoui, Celia Richardson, Emily Mabonga, Donna Ward, J. Francoise, W. Larbi, Sue Mitchell, A. Manning, V. Russell); Royal Free London Hospital, London (Fiona Burns [PI], Mark Harber, Nnenna Ngwu, Jonathan Edwards, Nargis Hemat, Tom Fernandez, Filippo Ferro, Jorge Ferreira, Alice Nightingale, Tasha Oakes-Monger, Darwin Matila, Pedro Nogueira, Victoria Mutagwanya); St. Georges University Hospitals, London (Catherine Cosgrove [PI], Lisa Hamzah, Catherine Emily Isitt, Helen Webb, Joyce Popoola, Kate Korley, Mark Mencias, Patricia Ribeiro, Rajeshwar Ramkhelawn, Sandra Oliva Lara, Sara Sajijad); Imperial College Healthcare NHS Trust, London (Alan Winston [PI], Jeremy Levy, Amber Shaw, Claire Petersen, Kyle Ring); University Hospital Lewisham, London (Melanie Rosenvinge [PI], Chloe Saad, Sarah Skinner, Thembi Moyo, Faith Odong, Katherine Gantert, Tina Ibe); Africa Advocacy Foundation (Denis Onyango); UK CHIC cohort (Caroline Sabin [PI], Teresa Hill)

## Disclosure

All the authors declared no competing interests.

## Supplementary Material

Supplementary File (PDF)

**Figure S1.** Proportion of participants with 2,1 and 0 *APOL1* variants.

**Figure S2.** Proportion of biopsy-confirmed cases of HIVAN/FSGS by African region.

**Table S1.** Baseline characteristics of study participants stratified by end-stage kidney disease and by renal impairment.

**Table S2.** Univariate association between *APOL1* high-risk genotypes and hypertension.

**Table S3.** Associated between *APOL1* high-risk genotypes and kidney outcomes.

**Table S3A.** Factors associated with ESKD (n = 99).

**Table S3B.** Factors associated with renal impairment (n = 221).

**Table S3C.** Factors associated with proteinuria (n=67ˆ).

**Table S3D.** Factors associated with albuminuria (n=99ˆ).

**Table S3E.** Factors associated with clinical diagnosis of HIVAN/FSGS/hypertensive nephropathy, n=19.

**Table S3F.** Factors associated with biopsy diagnosis of FSGS (excluding HIVAN), n=15.

**Table S3G.** Factors associated with biopsy diagnosis of HIVAN (excluding FSGS), n=37.

**Table S4.** Associations between *APOL1* risk variants and ESKD/renal impairment.

**Table S5.** Factors associated with *APOL1* high-risk genotypes and ESKD and renal impairment, inclusive of adjusting for hypertension.

**Table S6.** Factors associated with *APOL1* high-risk genotypes and ESKD and renal impairment, without correction factor for ethnicity in estimated GFR.

**Table S7.** Number and proportion of participants stratified by region of ancestry and APOL1 genotype.
