# Corrigendum to “Genetic Variants of *APOL1* Are Major Determinants of Kidney Failure in People of African Ancestry With HIV” [*Kidney International Reports* Volume 7, Issue 4, April 2022, Pages 786-796]

**DOI:** 10.1016/j.ekir.2024.10.011

**Published:** 2024-10-11

**Authors:** Rachel K.Y. Hung, Elizabeth Binns-Roemer, John W. Booth, Rachel Hilton, Mark Harber, Beatriz Santana-Suarez, Lucy Campbell, Julie Fox, Andrew Ustianowski, Catherine Cosgrove, James E. Burns, Amanda Clarke, David A. Price, David Chadwick, Denis Onyango, Lisa Hamzah, Kate Bramham, Caroline A. Sabin, Cheryl A. Winkler, Frank A. Post, GEN-AFRICA Study Group

**Affiliations:** 1 King’s College London, London, UK; 2 Basic Research Laboratory, Frederick National Laboratory for Cancer Research and the National Cancer Institute, Frederick, Maryland, USA; 3 Barts Health NHS Trust, London, UK; 4 Guy’s and St Thomas’ NHS Foundation Trust, London, UK; 5 Royal Free London Hospital NHS Foundation Trust, London, UK; 6 Pennine Acute Hospitals NHS Foundation Trust, Manchester, UK; 7 St George’s Hospital NHS Foundation Trust, London, UK; 8 University College London, London, UK; 9 Central and North West London NHS Foundation Trust, London, UK; 10 Brighton and Sussex University Hospital NHS Trust, Brighton, UK; 11 Brighton and Sussex Medical School Department of Infectious Disease, Brighton, UK; 12 The Newcastle Upon Tyne Hospitals, Newcastle, UK; 13 South Tees Hospitals NHS Foundation Trust, Middlesbrough, UK; 14 Africa Advocacy Foundation, London, UK; 15 King’s College Hospital NHS Foundation Trust, London, UK

The authors regret that the Supplementary Materials file was incomplete in the published article. The corrected Supplementary Materials file can be found using the Supplementary Materials link below.

