# Supplementary material for: Genetic Variants of APOL1 Are Major Determinants of Kidney Failure in People of African Ancestry With HIV
Source: Kidney Int Rep. 2022 Jan 25;7(4):786–96. doi: 10.1016/j.ekir.2022.01.1054 (PMC9039486; doi:10.1016/j.ekir.2022.01.1054)
Supplement: Supplementary File (PDF) [file mmc1.pdf]

Supplementary Material

Fig S1 Proportion of participants with 2,1 and 0 *APOL1* variants

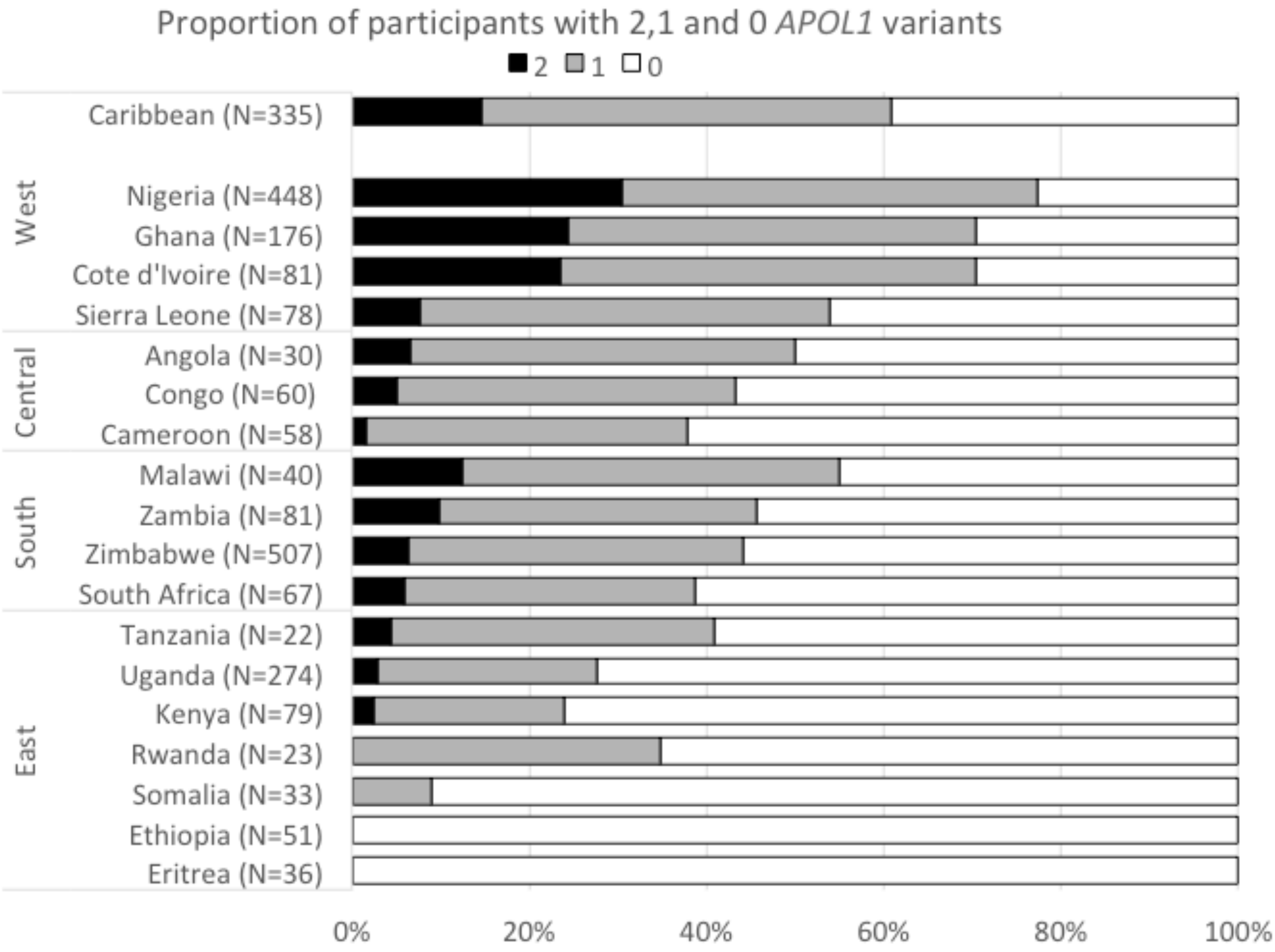

Fig S2 Proportion of biopsy-confirmed cases of HIVAN/FSGS by African region

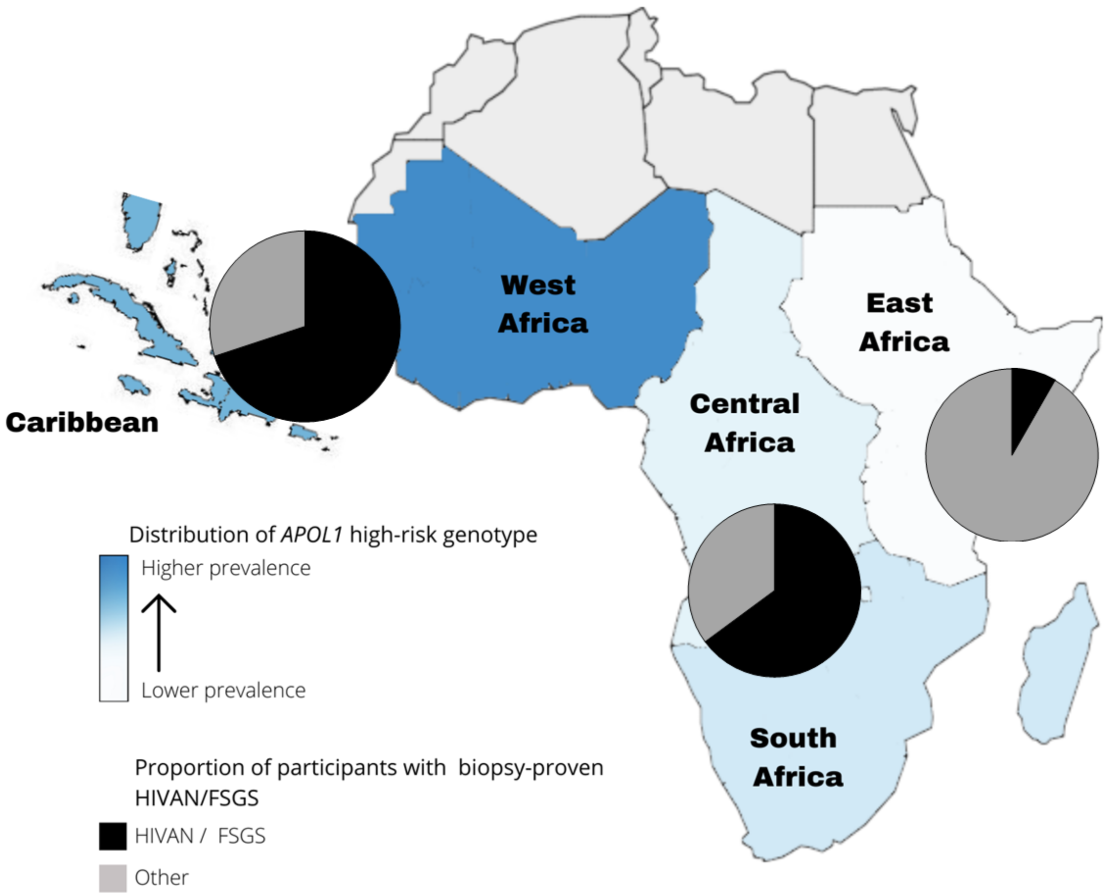

Distribution of *APOL1* genotype by African region

African Region		N	*APOL1* genotype	
			Low-risk	High-risk
East	n (%)	554	543 (98)	11 (2)
South	n (%)	765	713 (93)	52 (7)
Central	n (%)	157	148 (94)	9 (6)
West	n (%)	859	640 (75)	219 (25)
Caribbean	n (%)	347	297 (86)	50 (14)

Proportion of biopsy-confirmed HIVAN/FSGS by African region

African Region		N	Kidney Pathology	
			HIVAN/FSGS	Other
East	n (%)	12	1 (8)	11 (92)
South/Central	n (%)	17	11 (65)	6 (35)
West/Caribbean	n (%)	53	37 (70)	16 (30)
