# Supplementary material for: Corrigendum to “Genetic Variants of APOL1 Are Major Determinants of Kidney Failure in People of African Ancestry With HIV” [Kidney International Reports Volume 7, Issue 4, April 2022, Pages 786-796]
Source: Kidney Int Rep. 2024 Oct 11;9(12):3604. doi: 10.1016/j.ekir.2024.10.011 (PMC11652088; doi:10.1016/j.ekir.2024.10.011)
Supplement: Supplementary Material (PDF) [file mmc1.pdf]

Supplementary Material

Fig S1 Proportion of participants with 2,1 and 0 *APOL1* variants

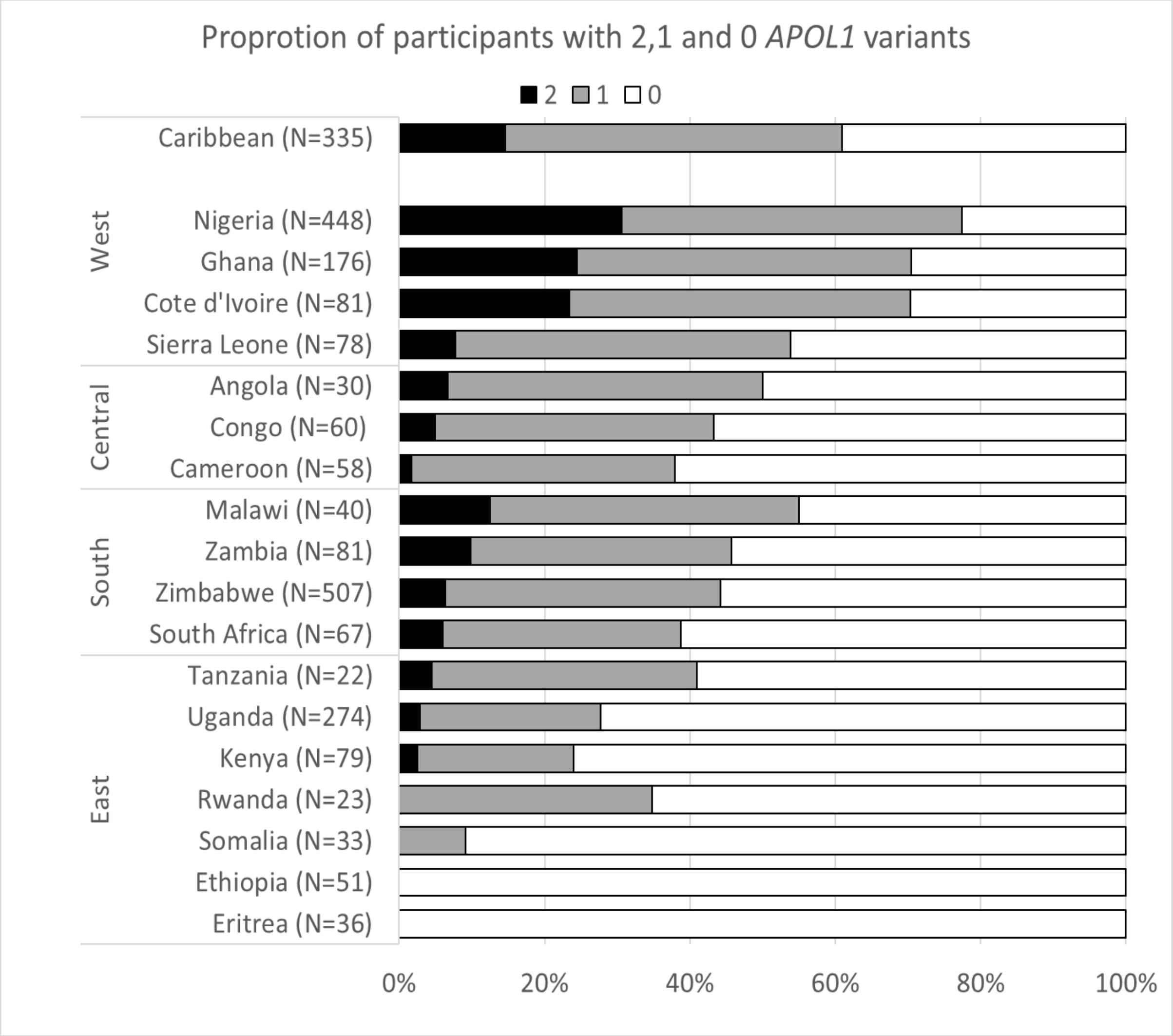

Fig S2 Proportion of biopsy-confirmed cases of HIVAN/FSGS by African region

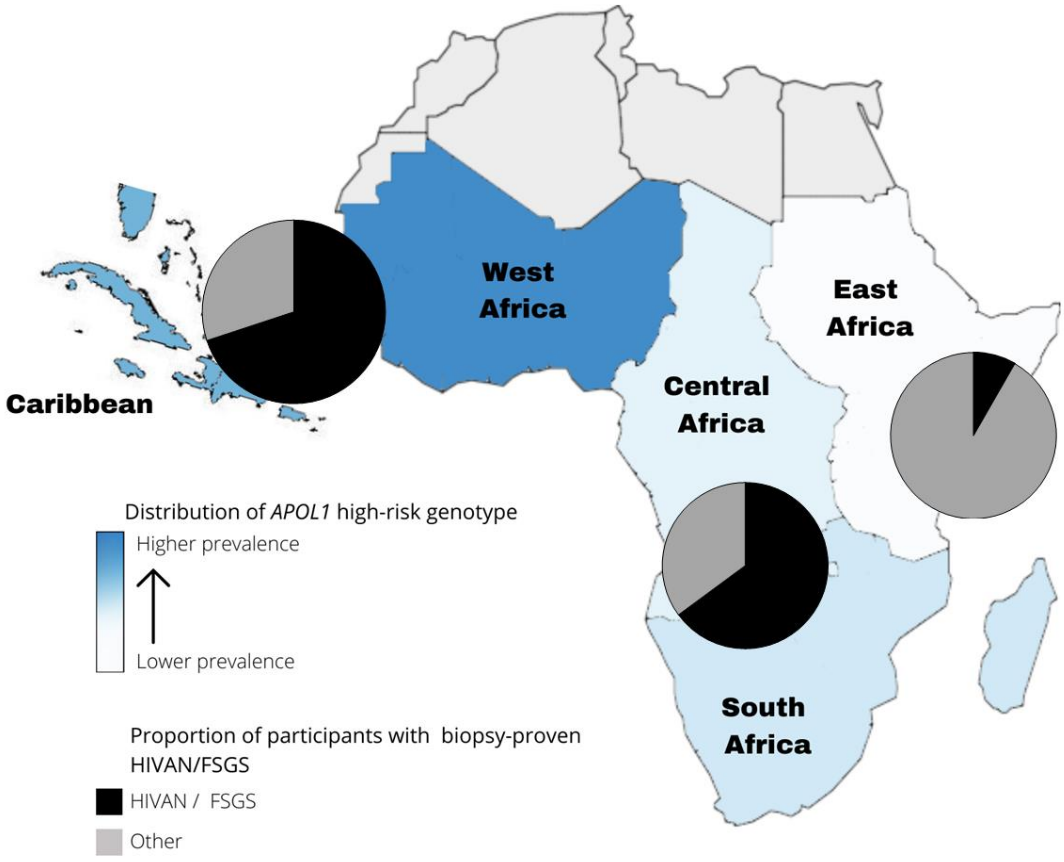

Distribution of *APOL1* genotype by African region

African Region		N	*APOL1* genotype	
			Low-risk	High-risk
East	n (%)	554	543 (98)	11 (2)
South	n (%)	765	713 (93)	52 (7)
Central	n (%)	157	148 (94)	9 (6)
West	n (%)	859	640 (75)	219 (25)
Caribbean	n (%)	347	297 (86)	50 (14)

Proportion of biopsy-confirmed HIVAN/FSGS by African region

African Region		N	Kidney Pathology	
			HIVAN/FSGS	Other
East	n (%)	12	1 (8)	11 (92)
South/Central	n (%)	17	11 (65)	6 (35)
West/Caribbean	n (%)	53	37 (70)	16 (30)

Table S1 Baseline characteristics of study participants stratified by end-stage kidney disease and by renal impairment

		Total	ESKD*	stage 1-4	p-value	eGFR <60	eGFR ≥60	p-value
		N=2,864	N=99	N=2,765		N=221	N=2,643	
Number of *APOL1* variants					<0.001			<0.001
0	n (%)	1,406 (49.1)	26 (26.3)	1,380 (49.9)		78 (35.3)	1,328 (50.2)	
1	n (%)	1,104 (38.5)	18 (18.2)	1,086 (39.3)		57 (25.8)	1,047 (39.6)	
2	n (%)	354 (12.4)	55 (55.6)	299 (10.8)		86 (38.9)	268 (10.1)	
Age, years	mean (sd)	48.1 (10.3)	50.6 (7.8)	48.0 (10.3)	0.013	53.9 (10.2)	47.6 (10.1)	<0.001
Sex, female	n (%)	1,641 (57.3)	44 (44.4)	1,597 (57.8)	0.008	104 (47.1)	1,537 (58.2)	<0.001
Region of ancestry					<0.001			<0.001
East Africa	n (%)	554 (19.3)	15 (15.2)	539 (19.5)		33 (14.9)	521 (19.7)	
South Africa	n (%)	765 (26.7)	13 (13.1)	752 (27.2)		41 (18.6)	724 (27.4)	
Central Africa	n (%)	157 (5.5)	5 (5.1)	152 (5.5)		9 (4.1)	148 (5.6)	
West Africa	n (%)	859 (30.0)	50 (50.5)	809 (29.3)		99 (44.8)	760 (28.8)	
Caribbean	n (%)	347 (12.1)	13 (13.1)	334 (12.1)		33 (14.9)	314 (11.9)	
Other	n (%)	182 (6.4)	3 (3.0)	179 (6.5)		6 (2.7)	176 (6.7)	
HIV mode of acquisition					<0.001			0.04
Heterosexual	n (%)	2,347 (81.9)	80 (80.8)	2,267 (82.0)		183 (82.8)	2,164 (81.9)	
MSM	n (%)	74 (2.6)	2 (2.0)	72 (2.6)		6 (2.7)	68 (2.6)	
Vertical	n (%)	232 (8.1)	1 (1.0)	231 (8.4)		8 (3.6)	224 (8.5)	
Blood products	n (%)	23 (0.8)	3 (3.0)	20 (0.7)		3 (1.4)	20 (0.8)	
Unknown	n (%)	188 (6.6)	13 (13.1)	175 (6.3)		21 (9.5)	167 (6.3)	
Time since HIV diagnosis, years	mean (sd)	14.0 (6.5)	14.3 (6.8)	14.0 (6.5)	0.57	14.8 (6.8)	13.9 (6.4)	0.063
Previous AIDS	n (%)	665 (24.0)	30 (31.6)	635 (23.7)	0.08	81 (37.7)	584 (22.8)	<0.001
Nadir CD4 cell count, cells/mm^3	mean (sd)	200 (80-340)	136 (37-225)	205 (82-343)	<0.001	119 (26-232)	210 (86-345)	<0.001
Recent CD4 cell count, cells/mm^3	mean (sd)	560 (403-735)	350 (258-512)	567 (411-740)	<0.001	443 (304-631)	569 (412-743)	<0.001
On antiretroviral therapy	n (%)	2,669 (93.2)	88 (88.9)	2,581 (93.3)	0.08	205 (92.8)	2,464 (93.2)	0.79
HIV RNA <200 copies/mL		2,832 (98.9)	98 (99.0)	2,734 (98.9)	0.92	218 (98.6)	2,614 (98.9)	0.72
HBsAg positive	n (%)	159 (5.6)	8 (8.4)	151 (5.5)	0.23	15 (7.1)	144 (5.5)	0.34
Anti-HCV positive	n (%)	35 (1.2)	5 (5.4)	30 (1.1)	<0.001	8 (3.8)	27 (1.0)	<0.001

Diabetes	n (%)	286 (10.1)	23 (23.2)	263 (9.6)	<0.001	56 (25.9)	230 (8.8)	<0.001
Hypertension	n (%)	915 (32.0)	94 (94.9)	821 (29.7)	<0.001	191 (86.4)	724 (27.4)	<0.001
Cardiovascular diseaseƚ	n (%)	123 (4.3)	19 (19.2)	104 (3.8)	<0.001	33 (14.9)	90 (3.4)	<0.001
BMI, kg/m^2					0.02			0.49
<18.5	n (%)	23 (0.8)	2 (2.1)	21 (0.8)		3 (1.4)	20 (0.8)	
18.5-24.9	n (%)	630 (22.4)	27 (28.7)	603 (22.2)		52 (24.5)	578 (22.2)	
25-29.9	n (%)	1,016 (36.1)	40 (42.6)	976 (35.9)		79 (37.3)	937 (36.0)	
≥30	n (%)	1,146 (40.7)	25 (26.6)	1,121 (41.2)		78 (36.8)	1,068 (41.0)	
Smoking status					0.03			0.007
Never	n (%)	2,218 (77.4)	72 (72.7)	2,146 (77.6)		170 (76.9)	2,048 (77.5)	
Ex	n (%)	321 (11.2)	19 (19.2)	302 (10.9)		36 (16.3)	285 (10.8)	
Current	n (%)	325 (11.3)	8 (8.1)	317 (11.5)		15 (6.8)	310 (11.7)	
uPCR^, mg/mmol								<0.001
<15	n (%)	2,193 (79.3)				92 (41.6)	2,149 (81.3)	
15-49	n (%)	431 (15.6)				58 (26.2)	396 (15.0)	
50-99	n (%)	74 (2.7)				28 (12.7)	55 (2.1)	
>=100	n (%)	67 (2.4)				43 (19.5)	43 (1.6)	
uACR^, mg/mmol								<0.001
<3	n (%)	2,189 (82.2)				48 (40.7)	2,141 (84.1)	
3-30	n (%)	375 (14.1)				31 (26.3)	344 (13.5)	
>30	n (%)	99 (3.7)				39 (33.1)	60 (2.4)	

*ESKD=eGFR <15mL/min/1.73m^2 and those on chronic dialysis or have had a kidney transplant
ƚCardiovascular disease = composite of any previous history of myocardial infarction, coronary artery disease, peripheral vascular disease, stroke, heart failure and cardiomyopathy
^ uPCR and uACR does not include participants with ESKD

ART=antiretroviral therapy; MSM=men who have sex with men; AIDS=acquired immunodeficiency syndrome; HBsAg=hepatitis B surface antigen; Anti-HCV=hepatitis C virus antibody; BMI=body mass index; uPCR=urine protein/creatinine ratio; uACR=urine albumin/creatinine ratio; ESKD=end-stage kidney disease; SD=standard deviation; IQR=interquartile range

Table S2 Univariate association between *APOL1* high-risk genotypes and hypertension

			Univariate		
**Number of *APOL1* variants**	**eGFR strata**		**OR**	**95% CI**	**p value**
2 (vs 0/1)	eGFR>90	N=1848	1.21	0.83 - 1.76	0.31
2 (vs 0/1)	eGFR 60-90	N=795	1.63	1.08 - 2.46	0.02
2 (vs 0/1)	eGFR <60	N=221	3.68	1.35 - 10.03	0.01

eGFR: estimated glomerular filtration rate in mL/min/1.73m^2; OR = odds ratio; 95% CI = 95% confidence interval

Table S3 Associated between *APOL1* high-risk genotypes and kidney outcomes

Table S3A: Factors associated with ESKD (n = 99)

	**Univariate**		**Adjusted for age, sex, ancestry**		**Additionally adjusted for HIV factors**		**Additionally adjusted for DM and CVD**	
	**OR (95% CI)**	**p value**	**OR (95% CI)**	**p value**	**OR (95% CI)**	**p value**	**OR (95% CI)**	**p value**
Number of *APOL1* variants								
0/1	1		1				1	
2	10.31 (6.81 - 15.60)	<0.001	9.84 (6.18 - 15.67)	<0.001	10.45 (6.21 - 17.61)		10.58 (6.22 - 17.99)	<0.001
Region of ancestry		<0.001*		0.32*		0.67*		0.72*
East Africa	1		1		1		1	
South Africa	0.62 (0.29 - 1.32)	0.21	0.48 (0.22 - 1.04)	0.06	0.5 (0.22 - 1.14)	0.10	0.52 (0.23 - 1.20)	0.13
Central Africa	1.18 (0.42 - 3.30)	0.75	0.96 (0.33 - 2.79)	0.95	0.68 (0.20 - 2.28)	0.53	0.67 (0.19 - 2.35)	0.53
West Africa	2.22 (1.253 - 4.00)	0.008	0.87 (0.45 - 1.70)	0.70	0.76 (0.37 - 1.56)	0.45	0.79 (0.38 - 1.67)	0.54
Caribbean	1.4 (0.66 - 2.98)	0.38	0.66 (0.29 - 1.49)	0.32	0.75 (0.31 - 1.78)	0.51	0.84 (0.35 - 2.05)	0.71
Other	0.6 (0.17 - 2.10)	0.43	0.49 (0.14 - 1.77)	0.28	0.54 (0.14 - 2.02)	0.36	0.58 (0.15 - 2.27)	0.43
Age (years)		0.02*		0.04*		0.08*		0.33*
<45	1		1		1		1	
45-54.9	1.88 (1.13 - 3.12)		1.8 (1.07 - 3.05)	0.03	1.73 (0.96 - 3.10)	0.07	1.58 (0.86 - 2.90)	0.14
>55	1.9 (1.09 - 3.30)		1.9 (1.06 - 3.42)	0.03	1.97 (1.04 - 3.76)	0.04	1.4 (0.70 - 2.81)	0.34
Sex (female vs. male)	0.58 (0.39 - 0.88)	0.009	0.68 (0.44 - 1.04)	0.07	0.89 (0.55 - 1.43)	0.63	0.88 (0.54 - 1.43)	0.61
Time since HIV diagnosis (per 5-year increment)	1.05 (0.90 - 1.22)	0.57						
Prior AIDS	1.49 (0.96 - 2.31)	0.08			1.43 (0.87 - 2.33)	0.16	1.2 (0.72 - 2.00)	0.49
Recent CD4 cell count, cells/mm^3		<0.001				<0.001*		<0.001*
$\geq$500	1				1		1	
350-499	2.11 (1.19 - 3.75)	0.01			2.09 (1.11 - 3.93)	0.02	2.25 (1.18 - 4.30)	0.01
<350	6.22 (3.87 - 10.01)	<0.001			5.76 (3.32 - 10.00)	<0.001	6.22 (3.52 - 10.98)	<0.001
On antiretroviral therapy	1.11 (0.15 - 0.22)	0.92						
HIV viral load (<200 copies/mL)	0.87 (0.30 - 1.09)	0.09			0.78 (0.38 - 1.60)	0.50	0.81 (0.39 - 1.71)	0.58

HBsAg positive	1.57 (0.75 - 3.30)	0.23				
Anti-HCV positive	5.15 (1.95 - 13.59)	0.001	3.73 (1.13 - 12.60)	0.03	3.01 (0.88 - 10.29)	0.08
Diabetes	2.85 (1.76 - 4.62)	<0.001			2.72 (1.44 - 5.12)	0.002
Hypertension	44.45 (18.01 - 109.67)	<0.001				
Cardiovascular disease	6.07 (3.55 - 10.39)	<0.001			5.9 (3.06 - 11.36)	<0.001

ESKD = eGFR <15 mL/min/1.73m^2 and those on chronic dialysis or have had a kidney transplant

Table S3B: Factors associated with renal impairment (n = 221)

	Univariate		Adjusted for age, sex, ancestry		Additionally adjusted for HIV factors		Additionally adjusted for DM and CVD	
	**OR (95% CI)**	**p value**	**OR (95% CI)**	**p value**	**OR (95% CI)**	**p value**	**OR (95% CI)**	**p value**
**Factors associated with eGFR <60mL/min/1.72m² (n=221)**								
Number of *APOL1* variants								
0/1	1		1		1		1	
2	5.65 (4.19 - 7.61)	<0.001	5.3 (3.78 - 7.32)	<0.001	5.1 (3.57 - 7.29)	<0.001	5.5 (3.81 - 7.95)	<0.001
Region of ancestry		<0.001*		0.14*		0.02*		0.03*
East Africa	1		1		1		1	
South Africa	0.89 (0.56 - 1.43)	0.64	0.86 (0.53 - 1.40)	0.56	0.95 (0.56 - 1.59)	0.83	0.93 (0.54 - 1.59)	0.79
Central Africa	0.96 (0.45 - 2.05)	0.92	0.93 (0.43 - 2.04)	0.86	0.91 (0.39 - 2.10)	0.82	0.87 (0.37 - 2.11)	0.79
West Africa	2.06 (1.37 - 3.10)	0.001	1.35 (0.86 - 2.11)	0.24	1.84 (1.12 - 3.02)	0.02	1.79 (1.07 - 3.00)	0.03
Caribbean	1.66 (1.00 - 2.74)	0.05	1.06 (0.62 - 1.81)	0.83	1.5 (0.84 - 2.65)	0.17	1.58 (0.88 - 2.84)	0.13
Other	0.54 (0.22 - 1.31)	0.17	0.55 (0.22 - 1.37)	0.20	0.7 (0.27 - 1.82)	0.46	0.73 (0.28 - 1.89)	0.52
Age (years)		<0.001*		<0.001*		<0.001*		<0.001*
<45	1		1		1		1	
45-54.9	2.09 (1.42 - 3.09)	<0.001	2.05 (1.37 - 3.06)	<0.001	1.72 (1.11 - 2.64)	0.01	1.58 (1.01 - 2.48)	0.05
>55	4 (2.72 - 5.89)	<0.001	4.32 (2.87 - 6.49)	<0.001	3.82 (2.47 - 5.91)	<0.001	2.85 (1.79 - 4.54)	<0.001
Sex (female vs. male)	0.64 (0.49 - 0.54)	0.001	0.77 (0.57 - 1.04)	0.09	0.95 (0.69 - 1.30)	0.74	0.95 (0.68 - 1.31)	0.74
Time since HIV diagnosis (per 5-year increment)	1.1 (0.99 - 1.27)	0.06			1.21 (1.07 - 1.37)	0.002	1.19 (1.05 - 1.35)	0.006
Prior AIDS	2.05 (1.53 - 2.74)	<0.001			2.06 (1.49 - 2.84)	<0.001	1.9 (1.36 - 2.64)	<0.001
Recent CD4 cell count, cells/mm³		<0.001*				<0.001*		<0.001*
≥500	1				1		1	
350-499	1.55 (1.08 - 2.21)	0.02			1.47 (0.99 - 2.17)	0.06	1.63 (1.09 - 2.44)	0.02
<350	3.09 (2.25 - 4.25)	<0.001			2.91 (2.01 - 4.21)	<0.001	3.12 (2.13 - 4.57)	<0.001
On antiretroviral therapy	0.81 (0.24 - 2.67)	0.72						
HIV viral load (<200 copies/mL)	0.93 (0.55 - 1.58)	0.79						
HBsAg positive	1.31 (0.75 - 2.27)	0.34						

Anti-HCV positive	3.75 (1.68 - 8.37)	0.001	2.83 (1.13 - 7.08)	0.03	2.46 (0.95 - 6.37)	0.07
Diabetes	3.64 (2.61 - 5.08)	<0.001			2.89 (1.93 - 4.32)	<0.001
Hypertension	16.85 (11.36 - 24.99)	<0.001				
Cardiovascular disease	4.97 (3.25 - 7.61)	<0.001			3.69 (2.25 - 6.03)	<0.001

Renal impairment = eGFR<60mL/min/1.73m^2

Table S3C: Factors associated with proteinuria (n=67^)

	Univariate	
	OR (95% CI)	p value
Number of *APOL1* variants		
0/1	1	
2	1.83 (0.97 - 3.46)	0.06
Region of ancestry		0.73*
East Africa	1	
South Africa	1.28 (0.64 - 2.54)	0.49
Central Africa	0.54 (0.12 - 2.42)	0.42
West Africa	0.97 (0.48 - 1.99)	0.94
Caribbean	0.74 (0.28 - 1.97)	0.55
Other	0.92 (0.30 - 2.87)	0.89
Age (years)		
<45	1	
45-54.9	1.37 (0.78 -2.40)	0.27
>55	1.08 (0.55 - 2.10)	0.83
Sex (female vs. male)	1.16 (0.70 - 1.90)	0.57
Time since HIV diagnosis (per 5 year increment)	0.97 (0.80 - 1.17)	0.76
Prior AIDS	2.28 (1.39 - 3.76)	0.001
Recent CD4 cell count, cells/mm^3		0.003*
>500	1	
350-499	0.41 (0.17 - 0.96)	0.04
<350	1.77 (1.03 - 3.05)	0.04
On antiretroviral therapy	-	
HIV viral load (<200 copies/mL)	1.53 (0.48 - 4.93)	0.47
HBsAg positive	0.26 (0.04 - 1.90)	0.19

Anti-HCV positive	-	
Diabetes	4.55 (2.64 - 7.84)	<0.001
Hypertension	3.63 (2.21 - 5.96)	<0.001
Prior cardiovascular disease	3.67 (1.70 - 7.90)	0.001

^does not include participants with ESKD (eGFR <15mL/min/1.73m^2 and those on chronic dialysis or have had a kidney transplant)

Proteinuria = urine protein creatinine ratio [PCR] >100mg/mmol

Table S3D: Factors associated with albuminuria (n=99^)

	Univariate		Adjusted for age, sex, ancestry		Additionally adjusted for HIV factors		Additionally adjusted for DM and CVD	
	**OR (95% CI)**	**p value**	**OR (95% CI)**	**p value**	**OR (95% CI)**	**p value**	**OR (95% CI)**	**p value**
Number of *APOL1* variants								
0/1	1		1		1		1	
2	2.57 (1.59 - 4.17)	<0.001	2.56 (1.58 - 4.16)	<0.001	2.85 (1.74 - 4.67)	<0.001	3.34 (2.00 - 5.56)	<0.001
Region of ancestry		0.21*						
East Africa	1							
South Africa	1.2 (0.61 - 2.36)	0.59						
Central Africa	1.83 (0.72 - 4.62)	0.2						
West Africa	1.95 (1.05 - 3.62)	0.03						
Caribbean	1.17 (0.51 - 2.66)	0.52						
Other	1.1 (0.39 - 3.06)	0.86						
Age (years)		0.03*		0.03*		0.18*		0.65*
<45	1		1		1		1	
45-54.9	1.73 (1.04 - 2.87)	0.03	1.69 (1.02 - 2.81)	<0.001	1.41 (0.83 - 2.38)	0.20	1.16 (0.67 - 2.00)	0.61
>55	1.97 (1.14 - 3.38)	0.02	1.94 (1.13 - 3.34)	<0.001	1.7 (0.96 - 2.99)	0.07	0.94 (0.50 - 1.77)	0.86
Sex (female vs. male)	0.77 (0.15 - 1.15)	0.2	0.83 (0.55 - 1.25)	0.97	0.82 (0.54 - 1.25)	0.36	0.85 (0.55 - 1.32)	0.48
Time since HIV diagnosis (per 5 year increment)	1.16 (0.99 - 1.35)	0.06			1.19 (1.02 - 1.39)	0.03	1.2 (1.02 - 1.41)	0.03
Prior AIDS	1.98 (1.30 - 3.02)	0.002			1.74 (1.13 - 2.68)	0.01	1.69 (1.08 - 2.64)	0.02
Recent CD4 cell count, cells/mm^3		0.06*				0.26*		
>500	1				1		1	
350-499	1.07 (0.63 - 1.80)	0.81			1.04 (0.61 - 1.78)	0.87	1.21 (0.70 - 2.09)	0.50
<350	1.81 (1.12 - 2.94)	0.02			1.7 (1.01 - 2.87)	0.04	1.7 (0.98 - 2.95)	0.06
On antiretroviral therapy	0.46 (0.11 - 1.97)	0.29						
HIV viral load (<200 copies/mL)	0.5 (0.27 - 0.92)	0.03			0.51 (0.27 - 0.99)	0.05	0.71 (0.47 - 1.05)	0.09
HBsAg positive	0.52 (0.16 - 1.66)	0.27						
Anti-HCV positive	-							

Diabetes	5.62 (3.61 - 8.77)	<0.001	5.36 (3.24 - 8.86)	<0.001
Hypertension	6.09 (3.92 - 9.47)	<0.001		
Prior cardiovascular disease	3.69 (1.90 - 7.16)	<0.001	2.91 (1.39 - 6.09)	0.005

^does not include participants with stage 5 CKD (eGFR <15mL/min/1.73m^2 and those on chronic dialysis or had a kidney transplant)

Albuminuria = urine albumin creatinine ratio [ACR] >30mmg/mmol

Table S3E: Factors associated with clinical diagnosis of HIVAN/FSGS/hypertensive nephropathy, n=19

	Univariate		Adjusted for age, sex, ancestry		Additionally adjusted for HIV factors		Additionally adjusted for DM and CVD	
	**OR (95% CI)**	**p value**	**OR (95% CI)**	**p value**	**OR (95% CI)**	**p value**	**OR (95% CI)**	**p value**
Number of *APOL1* variants								
0/1	1	<0.001	1		1		1	
2	14.64 (5.46 - 39.27)		14.34 (5.33 - 38.55)	<0.001	11.79 (4.18 - 33.30)	<0.001	12.77 (4.46 - 36.59)	<0.001
Age (years)		0.34*		0.41*		0.62*		0.61*
<45	1	0.17	1		1		1	
45-54.9	2.27 (0.71 - 7.25)	0.17	2.14 (0.66 - 6.91)	0.20	1.76 (0.51 - 6.14)	0.37	1.58 (0.45 - 5.61)	0.48
>55	1.48 (0.71 - 7.25)	0.53	1.51 (0.37 - 6.16)	0.57	1.17 (0.25 - 5.51)	0.85	0.87 (0.17 - 4.47)	0.87
Sex (female vs. male)	0.74 (0.29 - 1.88)		0.87 (0.34 - 2.23)	0.77	1.68 (0.58 - 4.85)	0.34	1.75 (0.60 - 5.09)	0.31
Recent CD4 cell count, cells/mm^3		0.002*				0.004*		0.003*
$\geq$500	1	0.06			1		1	
350-499	3.48 (0.93 - 12.98)	0.001			3.33 (0.73 - 15.31)	0.12	3.57 (0.78 - 16.53)	0.1
<350	7.49 (2.30 - 24.41)				8.48 (2.18 - 33.07)	0.002	9.26 (2.33 - 36.81)	0.002
		0.54						
Time since HIV diagnosis (per 5 year increment)	0.89 (0.62 - 1.29)	0.005						
Prior AIDS	4.12 (1.53 - 11.11)						3.43 (1.21 - 9.66)	0.02
On antiretroviral therapy	-	0.11						
HIV viral load (<200 copies/mL)	0.36 (0.10 - 1.26)	0.32						
HBsAg positive	2.11 (0.48 - 9.27)	0.13						
Anti-HCV positive	4.77 (0.62 - 36.87)				2.63 (0.28 - 24.89)	0.40	2.03 (0.21 - 19.72)	0.54
		0.1						
Diabetes	2.57 (0.84 - 7.87)	<0.001					2.77 (0.66 - 11.53)	0.16
Hypertension	36.82 (4.89 - 277.10)	0.79						
Prior cardiovascular disease	1.31 (0.17 - 9.94)						0.71 (0.08 - 6.13)	0.76

Table S3F: Factors associated with biopsy diagnosis of FSGS (excluding HIVAN), n=15

	Univariate		Adjusted for age, sex, ancestry		Additionally adjusted for HIV factors		Additionally adjusted for DM and CVD	
	**OR (95% CI)**	**p value**	**OR (95% CI)**	**p value**	**OR (95% CI)**	**p value**	**OR (95% CI)**	**p value**
Number of *APOL1* variants								
0/1	1		1		1		1	
2	11.81 (4.17 - 33.39)	<0.001	11.41 (4.02 - 32.43)	<0.001	16.78 (5.38 - 52.39)	<0.001	16.84 (5.29 - 53.64)	<0.001
Age (years)		0.38*		0.47*		0.91*		0.98*
<45	1		1		1		1	
45-54.9	2.12 (0.55 - 8.24)	0.28	1.91 (0.49 - 7.49)	0.35	1.06 (0.25 - 4.54)	0.94	0.93 (0.21 - 4.16)	0.93
>55	2.49 (0.59 - 10.45)	0.21	2.32 (0.54 - 9.89)	0.26	1.34 (0.29 - 6.23)	0.71	0.85 (0.16 - 4.61)	0.85
Sex (female vs. male)	0.27 (0.08 - 0.84)	0.02	0.31 (0.10 - 0.98)	0.05	0.36 (0.11 - 1.18)	0.09		
Recent CD4 cell count, cells/mm^3		0.23*						
≥500	1							
350-499	1.86 (0.52 - 6.61)	0.34						
<350	2.84 (0.86 - 9.33)	0.09						
Time since HIV diagnosis (per 5 year increment)	1.59 (1.10 - 2.30)	0.02			1.65 (1.14 - 2.40)	0.008	1.61 (1.11 - 2.36)	0.01
Prior AIDS	0.87 (0.24 - 3.14)	0.83						
On antiretroviral therapy	-							
HIV viral load (<200 copies/mL)	1.02 (0.13 - 7.80)	0.99						
HBsAg positive	-							
Anti-HCV positive	6.56 (0.83 - 51.66)	0.07			7.27 (0.77 - 69.06)	0.08	6.96 (0.74 - 65.36)	0.09
Diabetes	2.24 (0.63 - 7.98)	0.21					2.28 (0.53 - 9.79)	0.27
Hypertension	31.46 (4.13 - 239.65)	0.001						
Prior cardiovascular disease	8.68 (2.72 - 27.68)	<0.001					6.7 (1.88 - 23.94)	0.003

Table S3G: Factors associated with biopsy diagnosis of HIVAN (excluding FSGS), n=37

	**Univariate**		**Adjusted for age, sex, ancestry**		**Additionally adjusted for HIV factors**		**Additionally adjusted for DM and CVD**	
	**OR (95% CI)**	**p value**	**OR (95% CI)**	**p value**	**OR (95% CI)**	**p value**	**OR (95% CI)**	**p value**
Number of *APOL1* variants								
0/1	1		1		1		1	
2	24.49 (11.45 - 52.36)	<0.001	24.26 (11.32 - 52.0)	<0.001	30.89 (12.93 - 73.78)	<0.001	30.16 (12.48 - 72.88)	<0.001
Age (years)		0.27*		0.31		0.17*		
<45	1		1		1		1	
45-54.9	1.62 (0.71 - 3.68)	0.25	1.48 (0.64 - 3.42)	0.37	1.12 (0.43 - 2.94)	0.81	1.29 (0.48 - 3.44)	0.61
>55	1.99 (0.83 - 4.75)	0.12	2.01 (0.82 - 4.93)	0.13	2.31 (0.88 - 6.09)	0.09	2.68 (0.97 - 7.40)	0.06
Sex (female vs. male)	0.5 (0.26 - 0.97)	0.04	0.59 (0.30 - 1.16)	0.12	0.65 (0.30 - 1.40)	0.27	0.63 (0.29 - 7.40)	0.06
Recent CD4 cell count, cells/mm^3		<0.001*				<0.001*		<0.001*
$\geq$500	1				1		1	
350-499	1.95 (0.74 - 5.15)	0.18			2.31 (0.81 - 6.61)	0.12	2.18 (0.76 - 6.30)	0.15
<350	6.47 (2.99 - 13.99)	<0.001			6.01 (2.44 - 14.81)	<0.001	5.24 (2.11 - 13.00)	<0.001
Time since HIV diagnosis (per 5 year increment)	1.07 (0.83 - 1.37)	0.60						
Prior AIDS	1.81 (0.91 - 3.59)	0.09			1.86 (0.86 - 4.00)	0.12	1.83 (0.84 - 3.99)	0.13
On antiretroviral therapy	-							
HIV viral load (<200 copies/mL)	0.83 (0.25 - 2.71)	0.75						
HBsAg positive	2.18 (0.76 - 6.26)	0.15						
Anti-HCV positive	5.33 (1.22 - 23.19)	0.03			4.25 (0.68 - 26.34)	0.12	5.56 (0.90 - 34.31)	0.07
Diabetes	0.79 (0.24 - 2.59)	0.70					0.41 (0.08 - 2.02)	0.27
Hypertension	80.91 (11.07 - 591.05)	<0.001						
Prior cardiovascular disease	4.62 (1.89 - 11.30)	0.001					3.52 (1.25 - 9.93)	0.02
BMI, kg/m2		0.21						
<18.5	2.32 (0.29 - 18.66)	0.43						

18.5-24.9	1 ()	
25-29.9	0.61 (0.27 - 1.38)	0.24
>30	0.45 (0.19 - 1.05)	0.07

*p values are from the likelihood ratio test

AIDS=acquired immunodeficiency syndrome; HBsAg=hepatitis B surface antigen; Anti-HCV=hepatitis C virus antibody; uPCR=urine protein/creatinine ratio; uACR=urine albumin/creatinine ratio; eGFR = estimated glomerular filtration rate; FSGS = focal segmental glomerulosclerosis; HIVAN = HIV associated nephropathy; OR = odds ratio; 95% CI = 95% confidence interval

Table S4 Associations between *APOL1* risk variants and ESKD/renal impairment.

APOL1 variants	N	Univariable			Multivariable*		
		OR	95% CI	p value	OR	95% CI	p value
**ESKD[x]**							
G1G1 vs G0G0	29	10.4	5.97 - 18.13	<0.001	9.15	3.95 - 21.19	<0.001
G1G2 vs G0G0	22	10.71	5.88 - 19.53	<0.001	15.35	6.62 - 35.58	<0.001
G2G2 vs G0G0	4	5.05	1.69 - 15.13	0.004	8.29	2.19 - 31.48	0.002
G1G0 vs G0G0	14	1.17	0.61 - 2.26	0.63	1.08	0.49 - 2.40	0.86
G2G0 vs G0G0	4	0.47	0.16 - 1.35	0.16	0.34	0.09 - 1.25	0.11
G1G1 vs G2G2	29	2.06	0.68 - 6.18	0.20	1.25	0.33 - 4.78	0.75
G1G2 vs G2G2	22	2.12	0.69 - 6.51	0.19	2.02	0.48 - 8.50	0.34
G1G1 vs 0/1 variants	29	10.98	6.68 - 18.06	<0.001	8.75	4.52 - 16.94	<0.001
G1G2 vs 0/1 variants	22	11.31	6.55 - 19.54	<0.001	14.09	7.15 - 27.78	<0.001
G2G2 vs 0/1 variants	4	5.33	1.83 - 15.53	0.002	4.85	2.09 - 11.22	<0.001
**eGFR <60mL/min/1.73m^2**		**OR**	**95% CI**	**p value**			
G1G1 vs G0G0	45	5.8	3.86 - 8.73	<0.001	5.21	2.92 - 9.31	<0.001
G1G2 vs G0G0	33	5.73	3.63 - 9.04	<0.001	6.42	3.57 - 11.55	<0.001
G2G2 vs G0G0	8	3.58	1.62 - 7.94	0.002	5.53	2.25 - 13.60	<0.001
G1G0 vs G0G0	34	0.94	0.62 - 1.43	0.79	0.72	0.44 - 1.18	0.19
G2G0 vs G0G0	23	0.90	0.56 - 1.45	0.67	0.73	0.42 - 1.28	0.27
G1G1 vs G2G2	45	1.62	0.70 - 3.73	0.26	1.04	0.37 - 2.89	0.95
G1G2 vs G2G2	33	1.60	0.68 - 3.77	0.28	1.64	0.52 - 5.18	0.40
G1G1 vs 0/1 variants	45	6.00	4.10 - 8.77	<0.001	5.13	3.18 - 8.28	<0.001
G1G2 vs 0/1 variants	33	5.92	3.85 - 9.12	<0.001	6.30	3.78 - 10.48	<0.001
G2G2 vs 0/1 variants	8	3.70	1.69 - 8.09	0.001	7.18	2.19 - 23.52	0.001

[x]End-stage kidney disease (ESKD)=eGFR <15mL/min/1.73m^2 and those on chronic dialysis or have had a kidney transplant

*Multivariable model for ESKD adjusted for age, sex, African region of ancestry, prior AIDS, current CD4 cell count, hepatitis C virus antibody, diabetes, and cardiovascular disease; multivariable model for eGFR<60ml/min/1.73m^2 adjusted for the same covariates including time since HIV diagnosis (per 5-year increment).

eGFR = estimated glomerular filtration rate; OR = odds ratio; 95% CI = 95% confidence interval

Table S5 Factors associated with *APOL1* high-risk genotypes and ESKD and renal impairment, inclusive of adjusting for hypertension

	**Univariable**				**Multivariable^**			
	**OR**	**95% CI**	**p value**	**LRT p value**	**OR**	**95% CI**	**p value**	**LRT p value**
**A. Factors associated with ESKD[X], n=99**								
Number of *APOL1* variants								
0/1	1				1			
2	10.31	6.81 - 15.60	<0.001		7.32	4.16 - 12.86	<0.001	
Region of ancestry				<0.001				0.68
East Africa	1				1			
South Africa	0.62	0.29 - 1.32	0.21		0.5	0.21 - 1.19	0.12	
Central Africa	1.18	0.42 - 3.30	0.75		0.54	0.15 - 2.01	0.36	
West Africa	2.22	1.253 - 4.00	0.008		0.63	0.29 - 1.37	0.25	
Caribbean	1.40	0.66 - 2.98	0.38		0.84	0.33 - 2.13	0.72	
Other	0.60	0.17 - 2.10	0.43		0.72	0.17 - 3.04	0.66	
Age (years)								0.07
<45	1			0.02	1			
45-54.9	1.88	1.13 - 3.12			0.71	0.36 - 1.40	0.32	
>55	1.90	1.09 - 3.30			0.43	0.20 - 0.91	0.03	
Sex (female vs. male)	0.58	0.39 - 0.88	0.009		0.85	0.50 - 1.41	0.52	
Time since HIV diagnosis (per 5-year increment)	1.05	0.90 - 1.22	0.57					
Prior AIDS	1.49	0.96 - 2.31	0.08		0.92	0.53 - 1.59	0.76	
Recent CD4 cell count, cells/mm^3				<0.001				<0.001
≥500	1				1			
350-499	2.11	1.19 - 3.75	0.01		2.31	1.18 - 4.51	0.01	
<350	6.22	3.87 - 10.01	<0.001		6.32	3.47 - 11.51	<0.001	
On antiretroviral therapy	1.11	0.15 - 0.22	0.92					
HIV viral load (<200 copies/mL)	0.87	0.30 - 1.09	0.09		0.76	0.34 - 1.73	0.52	
HBsAg positive	1.57	0.75 - 3.30	0.23					
Anti-HCV positive	5.15	1.95 - 13.59	0.001		2.28	0.59 - 8.83	0.23	
Diabetes	2.85	1.76 - 4.62	<0.001		1.88	0.98 - 3.59	0.06	

Hypertension	44.45	18.01 - 109.67	<0.001		31.62	12.08 - 82.74	<0.001	
Cardiovascular disease	6.07	3.55 - 10.39	<0.001		3.39	1.70 - 6.74	0.001	
**B. Factors associated with eGFR <60 mL/min/1.73m^2 (n=221)**								
Number of *APOL1* variants				<0.001				
0/1	1				1			
2	5.65	4.19 - 7.61	<0.001		4.05	2.74 - 5.98	<0.001	
Region of ancestry				<0.001				0.08
East Africa	1				1			
South Africa	0.89	0.56 - 1.43	0.64		0.93	0.54 - 1.62	0.81	
Central Africa	0.96	0.45 - 2.05	0.92		0.78	0.32 - 1.91	0.59	
West Africa	2.06	1.37 - 3.10	0.001		1.58	0.92 - 2.72	0.1	
Caribbean	1.66	1.00 - 2.74	0.05		1.84	0.99 - 3.38	0.05	
Other	0.54	0.22 - 1.31	0.17		0.88	0.32 - 2.37	0.8	
Age (years)								
<45	1			<0.001	1			0.4
45-54.9	2.09	1.42 - 3.09	<0.001		0.97	0.60 - 1.57	0.9	
>55	4.00	2.72 - 5.89	<0.001		1.25	0.75 - 2.07	0.39	
Sex (female vs. male)	0.64	0.49 - 0.54	0.001					
Time since HIV diagnosis (per 5-year increment)	1.10	0.99 - 1.27	0.06		1.17	1.02 - 1.33	0.02	
Prior AIDS	2.05	1.53 - 2.74	<0.001		1.74	1.22 - 2.46	0.002	
Recent CD4 cell count, cells/mm^3				<0.001				<0.001
≥500	1				1			
350-499	1.55	1.08 - 2.21	0.02		1.71	1.13 - 2.60	0.01	
<350	3.09	2.25 - 4.25	<0.001		3.04	2.03 - 4.54	<0.001	
On antiretroviral therapy	0.81	0.24 - 2.67	0.72					
HIV viral load (<200 copies/mL)	0.93	0.55 - 1.58	0.79					
HBsAg positive	1.31	0.75 - 2.27	0.34					
Anti-HCV positive	3.75	1.68 - 8.37	0.001		2.36	0.82 - 6.79	0.11	
Diabetes	3.64	2.61 - 5.08	<0.001		1.99	1.31 - 3.02	0.001	
Hypertension	16.85	11.36 - 24.99	<0.001		9.33	6.04 - 14.41	<0.001	
Cardiovascular disease	4.97	3.25 - 7.61	<0.001		2.34	1.40 - 3.89	0.001	

[χ]ESKD=eGFR <15mL/min/1.73m^2 and those on chronic dialysis or had a kidney transplant
^Multivariable model adjusted for age, sex, African region of ancestry, time since HIV diagnosis (per 5-year increment), prior AIDS, current CD4 cell count, hepatitis C virus antibody, diabetes, cardiovascular disease, and hypertension

eGFR = estimated glomerular filtration rate; OR = odds ratio; 95% CI = 95% confidence interval

Table S6 Factors associated with *APOL1* high-risk genotypes and ESKD and renal impairment, without correction factor for ethnicity in estimated GFR

	**Univariate**		**Adjusted for age, sex, ancestry**		**Additionally adjusted for HIV factors**		**Additionally adjusted for DM and CVD**	
	**OR (95% CI)**	**p value**	**OR (95% CI)**	**p value**	**OR (95% CI)**	**p value**	**OR (95% CI)**	**p value**
**A. Factors associated with ESKD[X], n=102**								
Number of *APOL1* variants								
0/1	1		1		1		1	
2	9.64 (6.41 - 14.49)	<0.001	9.23 (5.84 - 14.58)	<0.001	9.56 (5.75 - 15.91)	<0.001	9.75 (5.80 - 16.39)	<0.001
Region of ancestry		0.0003		0.32		0.67		0.72
East Africa	1		1		1		1	
South Africa	0.63 (0.30 - 1.30)	0.21	0.50 (0.23 - 1.05)	0.07	0.52 (0.24 - 1.14)	0.10	0.54 (0.24 - 1.20)	0.13
Central Africa	1.11 (0.40 - 3.07)	0.85	0.91 (0.32 - 2.60)	0.86	0.65 (0.20 - 2.14)	0.48	0.63 (0.18 - 2.18)	0.46
West Africa	2.12 (1.20 - 3.76)	0.01	0.86 (0.45 - 1.65)	0.66	0.76 (0.38 - 1.53)	0.44	0.79 (0.39 - 1.63)	0.52
Caribbean	1.301 (0.62 - 2.76)	0.48	0.63 (0.28 - 1.40)	0.26	0.71 (0.30 - 1.66)	0.43	0.80 (0.33 - 1.90)	0.61
Other	0.56 (0.16 - 1.96)	0.37	0.46 (0.13 - 1.64)	0.23	0.51 (0.14 - 1.88)	0.31	0.55 (0.14 - 2.11)	0.39
Age (years)		0.02		0.05		0.09		
<45	1		1		1		1	0.39
45-54.9	1.85 (1.12 - 3.03)	0.02	1.75 (1.05 - 2.94)	0.03	1.67 (0.95 - 2.96)	0.08	1.50 (0.83 - 2.72)	0.18
>55	1.88 (1.09 - 3.25)	0.02	1.87 (1.06 - 3.33)	0.03	1.92 (1.03 - 3.60)	0.04	1.32 (0.67 - 2.61)	0.42
Sex (female vs. male)	0.58 (0.39 - 0.86)	0.007	0.66 (0.43 - 1.00)	0.05	0.85 (0.53 - 1.35)	0.49	0.84 (0.52 - 1.36)	0.48
Time since HIV diagnosis (per 5 year increment)	1.05 (0.91 - 1.23)	0.49						
Prior AIDS	1.49 (0.96 - 2.31)	0.08			1.43 (0.88 - 2.31)	0.15	1.20 (0.73 - 1.98)	0.48
Recent CD4 cell count, cells/mm^3		<0.001				<0.001		<0.001
≥500	1				1		1	
350-499	1.90 (1.09 - 3.34)	0.03			1.84 (0.99 - 3.41)	0.05	2.00 (1.07 - 3.75)	0.03
<350	5.61 (3.54 - 8.91)	<0.001			5.02 (2.95 - 8.55)	<0.001	5.45 (3.15 - 9.43)	<0.001
On antiretroviral therapy	1.15 (0.15 - 8.48)	0.89						
HIV viral load (<200 copies/mL)	0.59 (0.31 - 1.12)	0.11			0.79 (0.39 - 1.61)	0.52	0.83 (0.40 - 1.74)	0.62

HBsAg positive	1.57 (0.75 - 3.30)	0.23				
Anti-HCV positive	5.15 (1.95 - 13.59)	0.001	3.67 (1.14 - 11.88)	0.03	2.92 (0.87 - 9.85)	0.08
Diabetes	2.85 (1.76 - 4.62)	<0.001			3.01 (1.64 - 5.52)	<0.001
Hypertension	44.45 (18.01 - 109.67)	<0.001				
Cardiovascular disease	6.07 (3.55 - 10.39)	<0.001			5.56 (2.91 - 10.61)	<0.001

Table S6 Factors associated with *APOL1* high-risk genotypes and ESKD and renal impairment, without correction factor for ethnicity in estimated GFR

	Univariate		Adjusted for age, sex, ancestry		Additionally adjusted for HIV factors		Additionally adjusted for DM and CVD	
**B. Factors associated with renal impairment <60mL/min/1.72m^2, n=345**								
Number of *APOL1* variants								
0/1	1		1		1		1	
2	4.29 (3.30 - 5.57)	<0.001	4.15 (3.09 - 5.57)	<0.001	4.07 (2.94 - 5.46)	<0.001	4.18 (3.05 - 5.73)	<0.001
Region of ancestry		<0.001		0.05		0.02		0.02
East Africa	1		1		1		1	
South Africa	1.13 (0.77 - 1.65)	0.53	1.17 (0.79 - 1.74)	0.43	1.15 (0.76 - 1.73)	0.52	1.17 (0.77 - 1.77)	0.47
Central Africa	1.11 (0.61 - 2.05)	0.73	1.16 (0.61 - 2.19)	0.65	1.15 (0.59 - 2.23)	0.68	1.12 (0.57 - 2.20)	0.75
West Africa	2.05 (1.45 - 2.90)	<0.001	1.62 (1.11 - 2.37)	0.01	1.75 (1.18 - 2.59)	0.006	1.73 (1.16 - 2.59)	0.01
Caribbean	2.07 (1.37 - 2.12)	<0.001	1.45 (0.94 - 2.25)	0.09	1.69 (1.08 - 2.66)	0.02	1.75 (1.11 - 2.78)	0.02
Other	0.68 (0.34 - 1.34)	0.26	0.76 (0.38 - 1.52)	0.43	0.86 (0.42 - 1.76)	0.69	0.85 (0.40 - 1.79)	0.67
Age (years)		<0.001		<0.001		<0.001		<0.001
<45	1		1		1		1	
45-54.9	2.88 (2.04 - 4.05)	<0.001	2.85 (2.00 - 4.05)	<0.001	2.75 (1.90 - 3.98)	<0.001	2.63 (1.80 - 3.83)	<0.001
>55	6.22 (4.42 - 8.75)	<0.001	6.76 (4.73 - 9.67)	<0.001	6.92 (4.77 - 10.06)	<0.001	5.74 (3.89 - 8.46)	<0.001
Sex (female vs. male)	0.59 (0.47 - 0.74)	<0.001	0.72 (0.56 - 0.92)	0.009	0.81 (0.62 - 1.05)	0.10	0.80 (0.62 - 1.05)	0.10
Time since HIV diagnosis (per 5 year increment)	1.06 (0.97 - 1.16)	0.17						
Prior AIDS	1.82 (1.43 - 2.33)	<0.001			1.81 (1.38 - 2.36)	<0.001	1.66 (1.26 - 2.19)	<0.001
Recent CD4 cell count, cells/mm^3		<0.001				0.002		0.001
$\geq$500	1				1		1	
350-499	1.29 (0.96 - 1.71)	0.09			1.08 (0.79 - 1.48)	0.65	1.15 (0.83 - 1.58)	0.40
<350	2.18 (1.66 - 2.85)	<0.001			1.75 (1.28 - 2.39)	<0.001	1.84 (1.34 - 2.52)	<0.001
On antiretroviral therapy	0.74 (0.28 - 1.93)	0.53						
HIV viral load (<200 copies/mL)	0.84 (0.55 - 1.28)	0.42			0.92 (0.57 - 1.47)	0.72	0.92 (0.57 - 1.50)	<0.001

OR

HBsAg positive	1.57 (1.02 - 2.41)	0.04						
Anti-HCV positive	2.22 (1.00 - 4.94)	0.05			1.55 (0.64 - 2.74)	0.33	1.37 (0.55 - 3.41)	0.49
Diabetes	3.15 (2.35 - 4.22)	<0.001					2.08 (1.48 - 2.93)	<0.001
Hypertension	7.45 (5.78 - 9.59)	<0.001						
Cardiovascular disease	4.00 (2.70 - 5.94)	<0.001					2.94 (1.89 - 4.57)	<0.001

$^{\chi}$ESKD=eGFR <15mL/min/1.73m^2 and those on chronic dialysis or had a kidney transplant
eGFR = estimated glomerular filtration rate; OR = odds ratio; 95% CI = 95% confidence interval

Table S7 Number and proportion of participants stratified by region of ancestry and APOL1 genotype

A. All participants

		G0/G0	G1/G0	G2/G0	G1/G1	G1/G2	G2/G2	Total
		N=1,314	N=600	N=427	N=171	N=125	N=45	
Region of ancestry								
East	n (%)	432 (32.9)	50 (8.3)	61 (14.3)	1 (0.6)	6 (4.8)	4 (8.9)	554
South	n (%)	421 (32.0)	120 (20.0)	172 (40.3)	13 (7.6)	25 (20.0)	14 (31.1)	765
Central	n (%)	90 (6.8)	34 (5.7)	24 (5.6)	5 (2.9)	3 (2.4)	1 (2.2)	157
West	n (%)	237 (18.0)	287 (47.8)	116 (27.2)	128 (74.9)	72 (57.6)	19 (42.2)	859
Caribbean	n (%)	134 (10.2)	109 (18.2)	54 (12.6)	24 (14.0)	19 (15.2)	7 (15.6)	347

B. Participants with end-stage kidney disease

		G0/G0	G1/G0	G2/G0	G1/G1	G1/G2	G2/G2	Total
		N=25	N=13	N=4	N=29	N=21	N=4	
Region of ancestry								
East	n (%)	12 (80)	1 (7)	0 (0)	0 (0)	1 (7)	1 (7)	15
South	n (%)	6 (46)	2 (15)	1 (8)	0 (0)	2 (15)	2 (15)	13
Central	n (%)	2 (40)	1 (20)	0 (0)	1 (20)	1 (20)	0 (0)	5
West	n (%)	3 (6)	7 (14)	3 (6)	23 (46)	13 (26)	1 (2)	50
Caribbean	n (%)	2 (15)	2 (15)	0 (0)	5 (38)	4 (31)	0 (0)	13
